# Two-dimensional mesoporous materials: Synthetic strategies and emerging applications

**DOI:** 10.1016/j.isci.2026.117567

**Published:** 2026-09-24

**Authors:** Xirui Huang, Tiancong Zhao

**Affiliations:** 1 College of Chemistry and Materials, Department of Chemistry, Laboratory of Advanced Materials, Shanghai Key Laboratory of Molecular Catalysis and Innovative Materials, State Key Laboratory of Molecular Engineering of Polymers, Collaborative Innovation Center of Chemistry for Energy Materials (2011-iChEM), Fudan University, Shanghai 200433, China

**Keywords:** mesoporous, two-dimensional materials, self-assembly, nanomaterials, mesoporous materials

## Abstract

Two-dimensional (2D) mesoporous materials are an emerging class of porous nanostructures that combine mesoporosity with an ultrathin 2D geometry. Owing to their ultrathin geometry, high surface accessibility, shortened transport pathways, and anisotropic architectures, these materials have demonstrated exceptional promise in a broad range of applications, including catalysis, energy storage and conversion, sensing, and biomedicine. In this review, we provide a comprehensive overview of recent developments in 2D mesoporous materials, with particular emphasis on their synthetic principles, structural regulation strategies, and representative material systems. We further provide a critical analysis of representative applications by linking two-dimensional morphology, mesopore architecture, pore-wall characteristics, and secondary assembly to interfacial reactivity, biological interactions, and device-level performance. Finally, the key challenges associated with scalable synthesis, mechanistic understanding, structural regulation, and application-oriented design are discussed, together with future perspectives for advancing the next generation of 2D mesoporous materials.

## Graphical abstract

**Figure:**
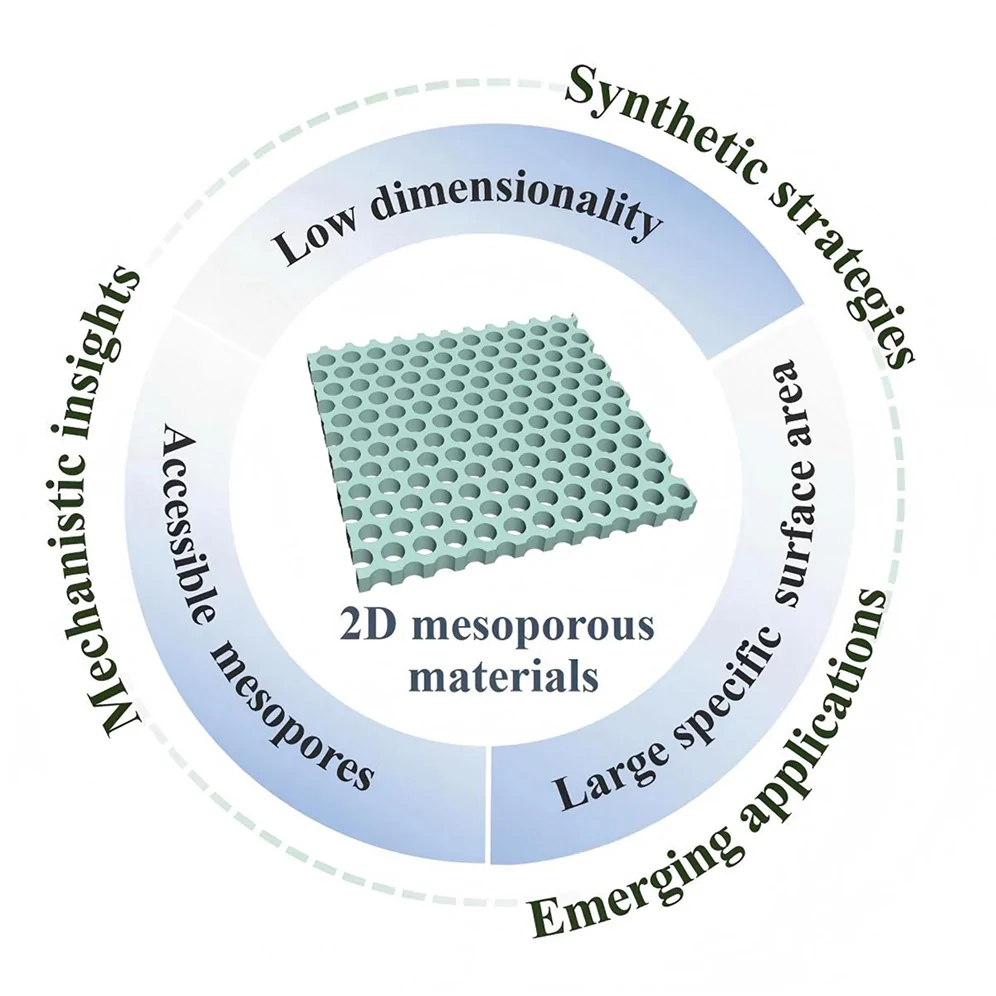

## Teaser

Applied sciences; Materials science; Materials synthesis

## Introduction

Over the past few decades, porous materials have demonstrated exceptional potential in fields including energy storage, biomedicine, chemical sensing, and catalysis, owing to their open framework structures, high specific surface areas, and tunable pore size distributions.^1^^,^^2^^,^^3^^,^^4^^,^^5^ According to the standards established by the International Union of Pure and Applied Chemistry (IUPAC), porous materials can be classified by pore diameter into microporous (<2 nm), mesoporous (2–50 nm), and macroporous (>50 nm) materials.^6^^,^^7^ Compared with microporous materials, which suffer from confined pore sizes, mesoporous structures offer improved mass transport.^8^^,^^9^^,^^10^ Meanwhile, in contrast to macroporous materials with relatively low specific surface areas, mesoporous materials provide abundant active sites and versatile platforms for interfacial reactions.^11^^,^^12^^,^^13^^,^^14^

Despite the significant advancements in traditional bulk mesoporous materials, their three-dimensional pore networks often suffer from long diffusion pathways, which limit their performance in various applications.^15^^,^^16^ In recent years, the emergence of two-dimensional (2D) mesoporous materials has provided a novel strategy to overcome these bottlenecks.^17^^,^^18^^,^^19^ By integrating abundant mesopores into nanosheets with nanoscale thicknesses and much larger lateral dimensions, the synergy between low-dimensional characteristics and mesoporous architectures endows these materials with a range of distinctive physicochemical properties, including structural anisotropy, highly accessible active sites, and enhanced mass-transport, mechanical, and electronic properties.^20^^,^^21^^,^^22^ However, fabricating 2D mesoporous materials is challenging because both dimensional confinement and mesostructure formation must be achieved simultaneously.^23^^,^^24^

For clarity, an inclusive, morphology-based operational definition of 2D mesoporous materials is adopted in this review. These materials should exist as identifiable sheet-like or platelet-like building blocks whose finite lateral boundaries belong to individual units rather than to a continuous macroscopic coating. Such boundaries may be generated directly during synthesis or inherited from discrete 2D templates. The materials should contain intrinsic mesopores of 2–50 nm and generally have a thickness of no more than approximately 100 nm. Their lateral dimensions should be clearly greater than their thickness, producing a pronounced anisotropic morphology. The morphological, thickness, and aspect-ratio criteria are applied together when determining whether a material falls within the scope of this review. Macroscopic films, membranes, electrodes, and interfacial coatings are not classified as discrete 2D mesoporous materials in this review. They are discussed separately in the context of device integration and practical applications. These device-level examples are not included in the material-level synthesis classification or structural comparison. For sandwich-like or retained-core heterostructures, mesoporosity refers to the integrated 2D building block and does not require every constituent layer to be independently mesoporous.

Within this defined scope, 2D mesoporous materials constitute a compositionally diverse but architecturally coherent class, encompassing silica-based and carbon-based materials, metals, metal oxides, framework materials, and sandwich-like composites (Figure 1). Their commonality lies not merely in a sheet-like appearance, but in the coexistence of a discrete nanoscale-thick 2D building block and intrinsic mesopores of 2–50 nm. Across these systems, synthesis must couple or appropriately sequence two fundamental processes: establishment of the finite 2D morphology and generation of the mesoporous network. Despite differences in precursors and framework-forming reactions, the formation of these 2D mesoporous structures can be understood based on several common principles. Accordingly, this review is organized around the origin of the mesopores and its coupling to 2D morphology formation, with focused discussion of representative material systems, composition-dependent synthetic constraints, and the resulting control over pore geometry, channel orientation, pore-wall structure, and sheet thickness. We further connect these structural attributes to interfacial reactivity, biological interactions, and device-level performance, thereby establishing transferable design principles across this chemically diverse yet structurally defined class of materials.

**Figure 1:**
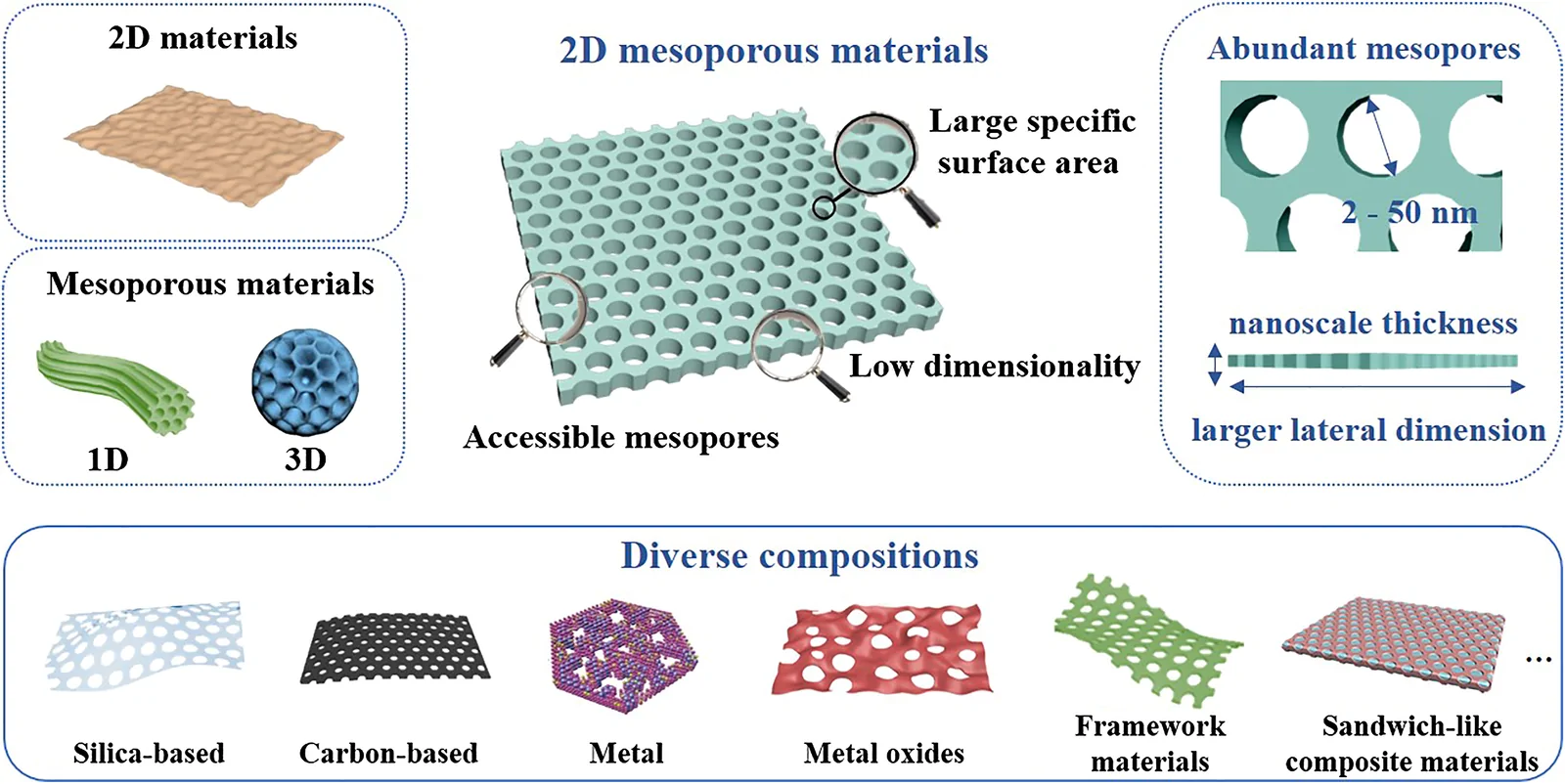
Scope, definition, and compositional diversity of two-dimensional (2D) mesoporous materials covered in this review

## Synthesis of two-dimensional mesoporous materials

In recent years, the synthetic methodologies for 2D mesoporous materials have expanded considerably, giving rise to diverse synthetic systems and approaches.^25^^,^^26^^,^^27^ Depending on whether the formation of the mesoporous framework relies on an ordered assembly process, synthetic strategies for 2D mesoporous materials can be broadly classified into two categories: assembly-mediated and reaction-derived.^28^^,^^29^^,^^30^ In assembly-mediated strategies, the mesoporous structure typically arises from the ordered arrangement of structure-directing units, such as surfactant micelles or block copolymers.^31^^,^^32^^,^^33^ Within this class, interface-induced assembly and directed assembly are distinguished according to whether the pore-forming units are spatially organized by a functionally distinct morphology-directing interface. In interface-induced assembly, this interface may be either pre-existing or generated *in situ*. It primarily defines the lateral geometry and suppresses out-of-plane growth, whereas separate micelles or block copolymers directly generate the mesopores. In directed assembly, no such functionally distinct interfacial platform is involved, and the 2D morphology and pore organization arise from the same self-assembling system or from its coupled structural transformations. Consequently, these methods usually enable the fabrication of 2D mesoporous materials with relatively high structural order.^34^ In contrast, reaction-derived strategies do not rely on preformed ordered assembly structures.^35^^,^^36^ In such systems, the mesoporous architecture generally originates from chemical reactions or structural evolution processes, such as gas release during reactions, selective consumption of specific components, or other related processes.^37^ As a result, mesoporous structures can form in the absence of assembly units, but they generally exhibit lower structural order. These representative strategies will be discussed in detail in the following sections. To provide a quantitative overview of the structural characteristics achieved using these synthetic strategies, Table 1 summarizes representative 2D mesoporous materials reported in recent years. The materials are classified according to their synthesis routes, together with the reported pore size, sheet thickness, and lateral dimensions. These parameters allow direct comparison of the accessible compositions and dimensional ranges across different synthetic systems. Because the extent of structural characterization varies among the original studies, values not explicitly reported are denoted as “not reported” (N.R.) rather than being inferred. Table 2 compares the four major strategies at the methodological level, focusing on their structural controllability, compositional flexibility, dependence on pre-existing interfaces or hosts, template-removal requirements, processing complexity, mesostructural order, and potential scalability.

**Table 1:** Representative 2D mesoporous materials, their synthesis routes, and reported structural parameters

Publication year	Materials	Synthesis method	Pore size	Thickness	Lateral dimensions	Reference
2018	ordered mesoporous carbon (OMC) sheets	hard interface-induced assembly	10 nm	∼9 nm	∼34 μm	Xi et al.^38^
2018	mesoporous metallic Ir nanosheets	directed assembly	∼15 nm	∼10 nm	A few hundred nanometers	Jiang et al.^39^
2018	single-layered 2D mesoporous TiO_2_ nanosheets	soft interface-induced assembly	∼4.0 nm	∼5.5 nm	∼0.5 μm	Lan et al.^40^
2018	mesoporous silicon nanosheets	post-synthetic formation	∼5.65 nm	<5 nm	μm-scale	Chen et al.^41^
2019	∗ZnS@SL-mTiO_2_; ∗GO@SL-mTiO_2_	hard interface-induced assembly	4.7–18.4 nm	∼11.5 nm	Template-dependent	Lan et al.^42^
2019	mesoporous IrO_*x*_ nanosheets	*in situ* formation	∼12–13 nm	∼10 nm	A few hundred nanometers	Jiang et al.^43^
2020	2D silica-cage superstructures	soft interface-induced assembly	N.R.	6–7 nm	μm-scale	Aubert et al.^44^
2020	mesoporous CeO_2_	hard interface-induced assembly	∼20 nm	15 nm	∼10 μm	Liu et al.^45^
2020	∗dual-mesoporous polypyrrole/graphene (DM-PG) nanosheets	hard interface-induced assembly	∼7 nm and 18 nm	∼80 nm	1–4 μm	Qin et al.^46^
2020	hierarchically porous carbon nanosheets	soft interface-induced assembly	25–46 nm	8.6–75 nm	μm-scale	Kim et al.^47^
2021	mesoporous Silicene@Silica	hard interface-induced assembly	2–3 nm	∼2.23 nm	∼200 nm	Yin et al.^48^
2022	Ir-IrO_*x*_/C layered nanosheets	directed assembly	∼10–30 nm	∼8.3–11 nm	N.R.	Zu et al.^49^
2022	∗mesoporous polyaniline/graphene (mPANI/G) nanosheets	hard interface-induced assembly	∼18 nm	∼30 nm	several micrometers	Wang et al.^50^
2023	BiOCl mesoporous single-crystalline nanosheets	*in situ* formation	3.4–6.4 nm	∼15 nm	>10 μm	Li et al.^51^
2023	mesoporous Ti_3_C_2_ MXene	post-synthetic formation	2–50 nm	intrinsic MXene thickness	a few hundred nanometers	Bu et al.^52^
2024	asymmetric monolayer mesoporous organosilica nanosheet	soft interface-induced assembly	∼20 nm	∼20 nm	μm-scale	Chen et al.^53^
2024	mesoporous Y_2_O_3_ nanosheet	*in situ* formation	5–10 nm	N.R.	μm-scale	Wang et al.^54^
2024	mesoporous SnO_2_	*in situ* formation	N.R.	6–8 nm	μm-scale	Jiang et al.^55^
2024	∗hierarchical porous covalent organic framework nanosheets	hard interface-induced assembly	7–40 nm	∼24 nm	μm-scale	Huang et al.^56^
2024	PtRhPb mesoporous nanosheets	post-synthetic formation	∼2–4 nm	5–8 nm	edge length 15–40 nm	Fan et al.^57^
2024	2D hierarchically porous UiO-66-NH_2_ nanosheets	soft interface-induced assembly	∼8 nm	∼28 nm	∼1.5 μm	Han et al.^58^
2024	N-doped 2D carbon nanosheets	soft interface-induced assembly	∼24 nm	∼10 nm	>0.5 μm	Lim et al.^59^
2024	∗Meso-holey carbon	hard interface-induced assembly	∼13.5 nm	∼5 nm	a few hundred nanometers	Kim et al.^34^
2024	2D Co-Cu mesoporous nanoplates	*in situ* formation	∼26 nm	∼75 nm	width ∼630 nm length ∼1750 nm	Han et al.^60^
2025	∗hierarchical mesoporous carbon/MXene nanosheets	hard interface-induced assembly	∼38 nm	8.0–29 nm	∼5 μm	Han et al.^61^
2025	mesoporous multimetal spinel supracrystals	hard interface-induced assembly	∼2.4 nm	∼8 nm	∼2.4 μm	Gao et al.^62^
2025	mesoporous N-doped carbon	soft interface-induced assembly	7.3–23.2 nm	∼10 nm	∼0.5 μm	Qin et al.^63^
2025	porous indium oxide nanosheets	*in situ* formation	∼2–20 nm	∼4.0 nm	μm-scale	Li et al.^64^
2025	∗mesoporous C-MXene-C heterostructure	hard interface-induced assembly	∼7 nm	∼21 nm	few-hundred nm-μm scale	Zhang et al.^65^
2025	mesoporous perovskite oxide nanosheets	*in situ* formation	∼2–50 nm	∼32.5 nm	μm-scale	Wang et al.^66^
2026	2D mesoporous silica nanodisks	directed assembly	∼10 nm	∼7 nm	0.2–1.0 μm	Dong et al.^67^
2026	mesoporous sulfonic silica nanosheets	soft interface-induced assembly	∼4.0 nm	∼8.5 nm	μm-scale	Wu et al.^68^
2026	mesoporous polydopamine (mPDA) nanosquares	hard interface-induced assembly	8–20 nm	14–25 nm	100–200 nm	Liu et al.^69^

Material names marked with an asterisk (∗) denote sandwich-like architectures in which a retained 2D core or support is integrated with mesoporous layers in the final heterostructure. OMC, ordered mesoporous carbon; SL-mTiO_2_, single-layer mesoporous TiO_2_; GO, graphene oxide; DM-PG, dual-mesoporous polypyrrole/graphene; mPANI/G, mesoporous polyaniline/graphene; mPDA, mesoporous polydopamine; C–MXene–C, carbon/MXene/carbon; N.R., not reported. MXene denotes a family of two-dimensional transition-metal carbides, nitrides, and carbonitrides.

**Table 2:** Comparison of the main synthesis methods for 2D mesoporous materials

Synthesis method	Schematic diagram	Advantages	Disadvantages
**Assembly-mediated**	**(ⅰ) interface-induced assembly**	effective 2D confinement tunable channel orientation and layer number uniform and accessible mesostructures	interface sensitivity possible non-removable interface templates

	**(ⅱ) directed assembly**	no pre-existing 2D substrate simplified or one-pot processing flexible composition and pore design	coupled control of 2D shape and mesostructure narrow assembly window

**Reaction-derived**	**(ⅰ) post-synthetic formation**	inheritance of preformed 2D morphology broad material applicability	preformed sacrificial domains required multistep processing and material loss limited pore order and possible framework damage

	**(ⅱ) *in situ* formation**	concurrent formation of 2D morphology and porosity no pre-existing 2D host required potentially rapid and scalable processing	strong kinetic dependence limited independent structural control often low pore order and morphology uniformity

Taken together, Tables 1 and 2 provide complementary material-level and strategy-level perspectives on the synthesis of 2D mesoporous materials. Building on this comparative overview, the following sections discuss interface-induced assembly, directed assembly, post-synthetic pore formation, and *in situ* porous-sheet formation individually, with particular emphasis on their formation mechanisms, representative material systems, structural controllability, and practical limitations.

### Interface-induced assembly strategy

Interface-induced assembly is one of the most established and controllable strategies for the synthesis of 2D mesoporous materials.^70^^,^^71^ In this approach, a spatially extended morphology-directing interface guides precursor growth and suppresses out-of-plane extension, thereby defining the lateral geometry of the resulting 2D architecture.^72^ Importantly, the interfacial carrier does not directly generate the mesopores. The mesoporous structure is instead produced by separate pore-forming units, such as surfactant micelles or block copolymers, assembled at the interface.^73^ Here, the term “interfacial template” therefore refers to a morphology-directing carrier and should not be confused with the micellar or block-copolymer template responsible for pore formation. Based on their interfacial stability and mechanical rigidity, such interfaces can generally be classified as “hard” or “soft”.^1^ Both types of interfaces are capable of providing effective spatial guidance for the formation of 2D architectures and have been widely applied in the construction of 2D mesoporous materials.^74^^,^^75^

Historically, hard-interface-induced mesostructural assembly originated from the synthesis of substrate-supported mesoporous thin films. In the late 1990s, Brinker and co-workers pioneered evaporation-induced self-assembly (EISA), in which solvent evaporation drove the cooperative organization of surfactants and inorganic precursors on planar solid substrates, enabling the rapid formation of continuous mesoporous silica films with ordered mesophases.^76^ This principle was subsequently extended to glass, silicon wafers, metallic foils, and other macroscopic planar substrates. Although these studies established the fundamental basis for interfacial confinement and mesostructural control, the resulting coatings are classified here as mesoporous thin films rather than 2D mesoporous materials. This classification remains unchanged when a continuous film can be scraped, fractured, transferred, or detached from its substrate, because such post-synthetic operations do not establish an intrinsic, laterally discrete building-block identity. Building on these thin-film precedents, subsequent studies shifted the morphology-directing interface from spatially continuous macroscopic substrates to discrete solid carriers with well-defined lateral dimensions. Such carriers preserve the planar confinement required for interfacial assembly while additionally defining the finite lateral boundaries of the resulting mesoporous architectures. Consequently, the assembled mesoporous layers can be released as identifiable freestanding sheets or retained as discrete 2D heterostructures.

“Hard” interfacial templates are typically solid materials featuring a two-dimensional planar surface, with fixed morphology and high structural stability. Examples include 2D transition-metal carbides, nitrides, and carbonitrides, collectively known as MXenes, graphene oxide (GO) nanosheets, layered metal hydroxides, as well as other sheet-like nanomaterials or planar substrates.^77^ These templates provide direct spatial guidance, enabling the formation of 2D nanosheets through growth on their surfaces. Their planar geometry generally remains unchanged throughout the reaction process. For instance, Tang et al. constructed a 2D mesoporous polypyrrole/reduced graphene oxide (mPPy/rGO) heterostructure (Figure 2A).^78^ Ultrathin GO nanosheets were used as a 2D interfacial template, and poly(ethylene oxide)-block-poly(propylene oxide)-block-poly(ethylene oxide) (PEO_20_-b-PPO_70_-b-PEO_20_) triblock copolymer (Pluronic P123) served as the structure-directing agent. A monolayer polypyrrole with cylindrical mesopores was precisely assembled on the GO sheets, with an average pore diameter of approximately 8 nm. This versatile strategy is extensible to various polymeric materials, where the orientation of the mesopores can be finely tuned by adjusting assembly conditions. Similarly, Wang et al. employed graphene as a 2D interface to synthesize polyaniline (PANI) nanosheets with controllable and uniform mesopores.^50^ The mesoporous PANI layers were grown on both sides of the graphene template, exhibiting a high specific surface area. Furthermore, the application of 2D interfacial templates has expanded significantly beyond carbon-based materials to include diverse inorganic substrates. Xi et al. utilized MgAl-layered double hydroxide (MgAl-LDH) to construct ordered mesoporous carbon nanosheets (Figure 2B), while Han et al. and Fang et al. successfully employed MXene and MoS_2_, respectively, to steer the assembly of mesoporous carbon heterostructures (Figures 2C and 2D).^38^^,^^61^^,^^79^^,^^80^ Moving beyond carbon compositions, Yin et al. synthesized mesoporous silica (mSiO_2_) using silicene nanosheets as the functional interface (Figure 2E).^48^ Furthermore, Lan et al. demonstrated the universality of this approach by developing a generalized strategy to coat mesoporous TiO_2_ shells onto various nanomaterials, including GO and ZnS (Figure 2F).^42^

**Figure 2:**
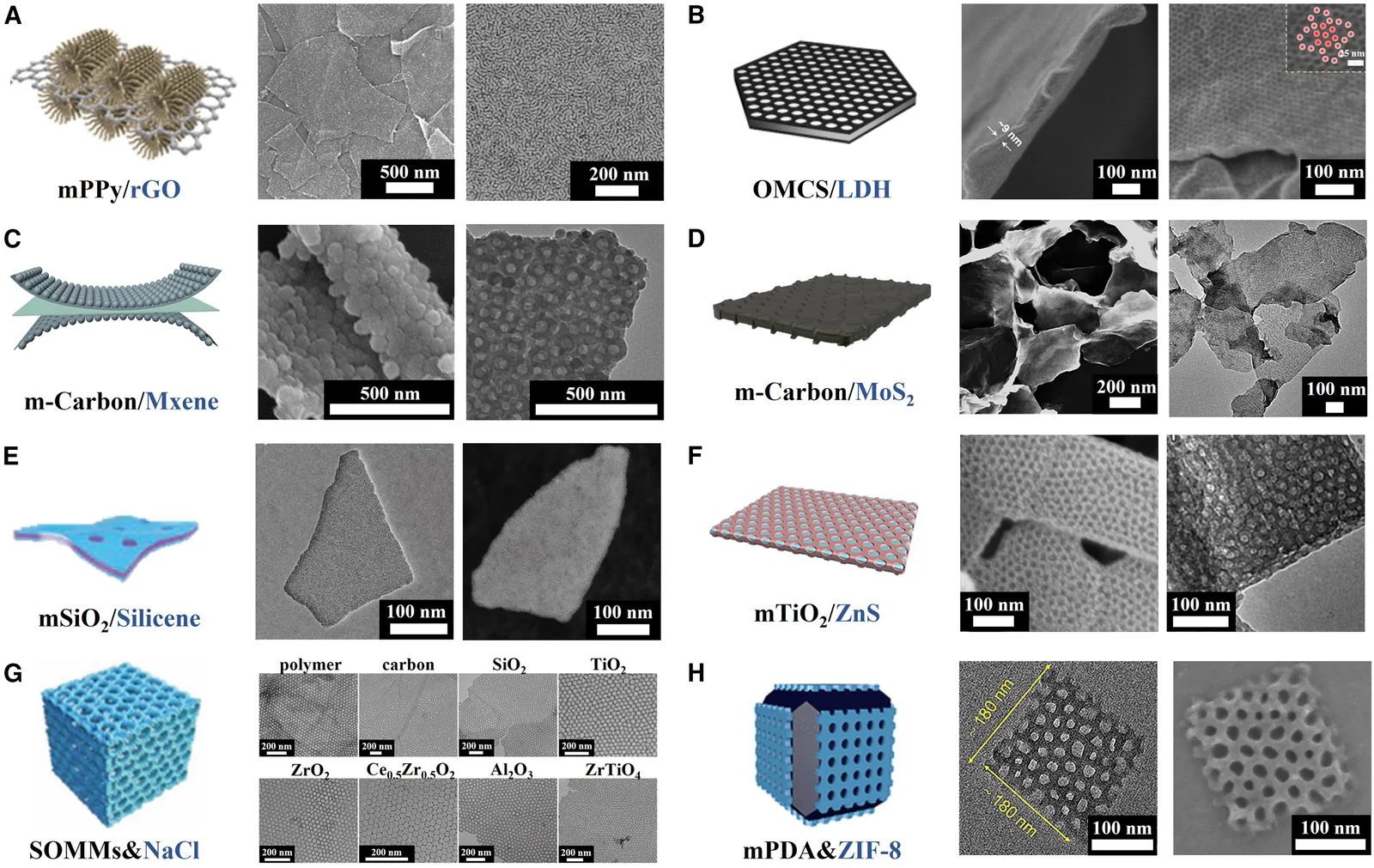
Synthesis of various 2D mesoporous materials assisted by “hard” interfaces (A) Mesoporous polypyrrole (mPPy)/reduced graphene oxide (rGO). Reproduced with permission from.^78^ Copyright © 2025 The Author(s). (B) Ordered mesoporous carbon sheets (OMCS)/layered double hydroxide (LDH). Reproduced with permission from.^38^ Copyright © 2018 American Chemical Society. (C) Mesoporous carbon/MXene. Reproduced with permission from.^61^ Copyright © 2025 Elsevier Inc. (D) Mesoporous carbon/MoS_2_. Reproduced with permission from.^79^ Copyright © 2016 Wiley-VCH Verlag GmbH & Co. KGaA, Weinheim. (E) Mesoporous silica (mSiO_2_)/silicene. Reproduced with permission from.^48^ Copyright © 2021 Wiley-VCH GmbH. (F) Mesoporous TiO_2_ (mTiO_2_)/ZnS. Reproduced with permission from.^42^ Copyright © 2019 Elsevier Inc. (G) Single-layer mesoporous materials (SOMMs)/NaCl. Reproduced with permission from.^45^ Copyright © 2020 Wiley-VCH Verlag GmbH & Co. KGaA, Weinheim. (H) Mesoporous polydopamine (mPDA)/zeolitic imidazolate framework-8 (ZIF-8). Reproduced with permission from.^69^ Copyright © 2026 American Chemical Society. Scale bars: (A) 500 nm (left) and 200 nm (right); (B) 100 nm in both images; (C) 500 nm in both images; (D) 200 nm (left) and 100 nm (right); (E) 100 nm in both images; (F) 100 nm in both images; (G) 200 nm in all images; and (H) 100 nm in both images.

The fundamental principle of the interfacial-confined assembly strategy is to provide a stable interfacial environment; therefore, interfacial templates are not limited to 2D nanomaterials. Salt nanoparticles and other materials with three-dimensional structures also hold potential as hard templates, since parts of their surfaces can act as interfacial carriers. The research group of Yonghui Deng at Fudan University proposed the concept of a “lab-on-salt-particle approach”, which enables the universal synthesis of various single-layer mesoporous materials, including mesoporous carbon, mesoporous SiO_2_, and mesoporous metal oxides (Figure 2G).^45^ In this synthetic system, a variety of structure-directing agents can be employed, including commercially available PEO_100_-b-PPO_65_-b-PEO_100_ triblock copolymer Pluronic F127 as well as laboratory-synthesized block copolymers such as poly(ethylene oxide)-block-polystyrene (PEO-b-PS) and polystyrene-block-poly(4-vinylpyridine) (PS-b-P4VP). Meanwhile, different molecular precursors can co-assemble with these structure-directing agents in various organic solvents. On the surface of the salt substrate, closely packed monomicelles can form a single-layer assembly, and the substrate can be removed simply by washing with pure water without destroying the ordered mesoporous structure. Building upon this concept, recent studies have further advanced the structural controllability of such systems. In particular, as increasingly precise control over the assembly process has been achieved, the focus has gradually shifted from universal interfacial platforms toward more deliberately engineered templates capable of directing anisotropic growth and regulating in-plane structural features. Recently, Liu et al. proposed an interface-confined anisotropic assembly strategy to synthesize square-shaped 2D polydopamine (PDA) nanosheets with mesoporous structures, in which both the lateral dimensions and the in-plane mesopore sizes can be tuned (Figure 2H).^69^ This strategy employs truncated rhombic dodecahedral ZIF-8 metal-organic framework (MOF) nanoparticles as hard templates to guide the selective assembly of micelles. Following etching of the ZIF-8 seeds, the resulting nanosheets preserve their well-defined square-shaped 2D morphology and mesoporous architecture.

An important practical distinction within hard interface-induced assembly concerns the fate of the interfacial carrier after assembly. When the carrier is sacrificial or removable, it can be dissolved, selectively etched, or delaminated to release a freestanding 2D mesoporous sheet. By contrast, when the original 2D carrier cannot be removed without disrupting the product or is deliberately retained, it becomes an integral component of the final supported heterostructure. In particular, mesoporous layers grown on both sides of a retained 2D core produce a sandwich-like architecture.^81^ Template removal or retention therefore directly determines the final morphology and composition of the product and further affects surface accessibility, diffusion pathways, mechanical integrity, electrical connectivity, and the range of potential applications.

These findings demonstrate that the introduction of an interface can serve as an ideal platform for constructing well-defined mesoporous architectures, while enabling precise regulation of nanosheet morphology, pore ordering, and mesostructural orientation.^82^ Moreover, the broad compatibility of this strategy with carbons, polymers, and metal oxides underscores its universal applicability across diverse materials systems.

Soft interfacial templates provide flexible and adaptable growth platforms for the synthesis of 2D mesoporous materials.^40^^,^^83^ Such interfaces include molecular bilayers, internal interfaces generated by phase separation between immiscible polymers, and liquid–liquid emulsion interfaces. Unlike rigid solid carriers, these dynamically formed interfaces can localize precursor assembly and impose anisotropic growth while retaining substantial compositional and structural adaptability.^15^^,^^83^ The research group of Yusuke Yamauchi successfully constructed ultrathin 2D mesoporous UiO-66 nanosheets by assembling polystyrene-block-poly(ethylene oxide) (PS-b-PEO) spherical micelles onto the surface of an amphiphilic perfluorooctanoic acid (PFCA) bilayer (Figure 3A).^84^ Moreover, similar strategies have been extended to the synthesis of diverse mesoporous polymers.^85^ Specifically, the research group of Shaohua Liu utilized PFCA bilayers and PS-b-PEO to form freestanding 2D bilayers, enabling the preparation of 2D polypyrrole (PPy) and PANI nanosheets with highly ordered mesopores.^86^ In these PFCA-based systems, the PFCA bilayer acts as a functionally distinct morphology-directing interface rather than a pore-forming template. Its structure does not directly generate the mesopores, which are instead defined by the PS-b-PEO micelles assembled at the interface. These routes are therefore classified as interface-induced assembly.

**Figure 3:**
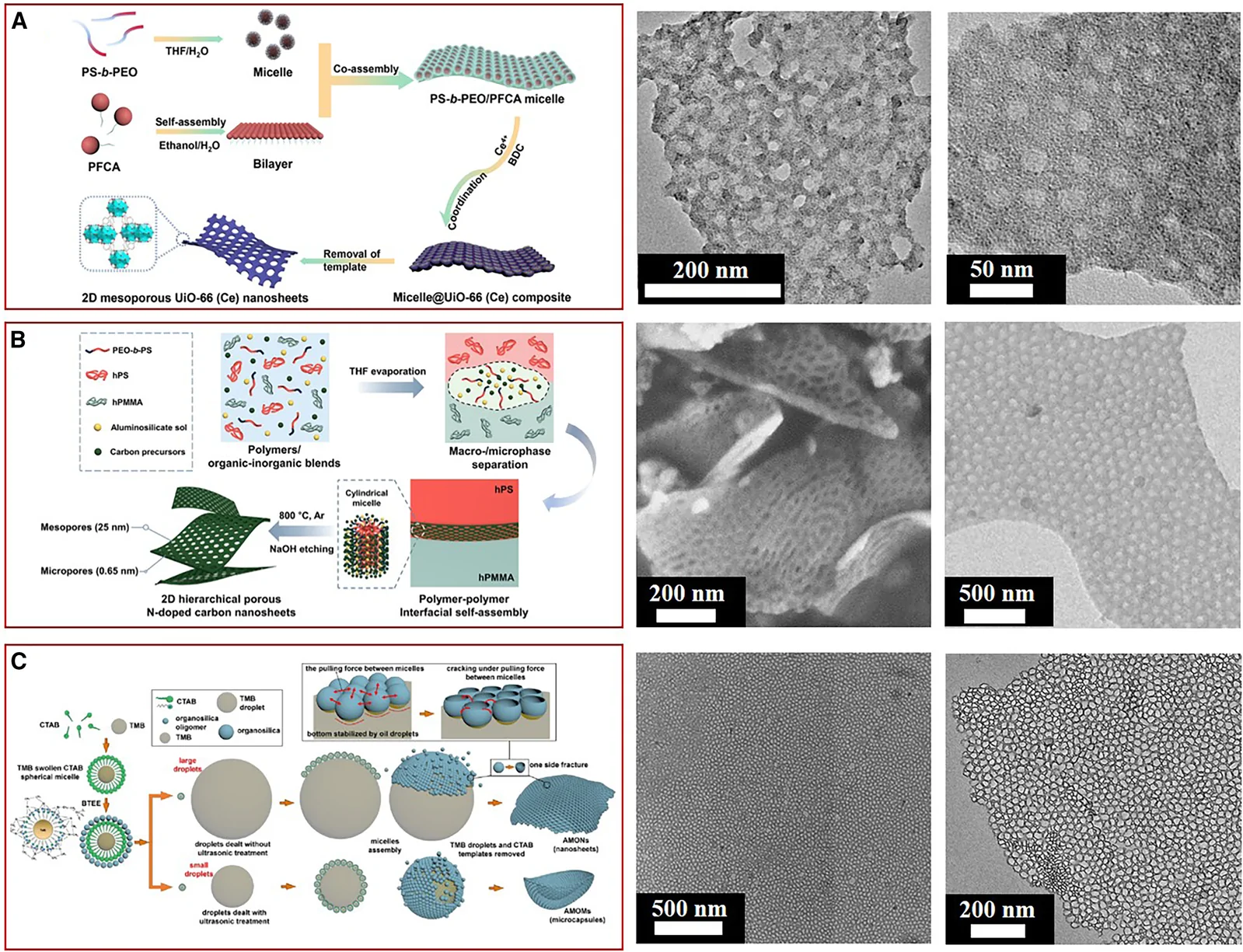
Synthesis of various 2D mesoporous materials assisted by “soft” interfacial templates (A) Use of a perfluorooctanoic acid (PFCA) bilayer to construct 2D mesoporous UiO-66 nanosheets. Reproduced with permission from.^84^ Copyright © 2025 The Author(s). (B) Polymer-polymer interfacial self-assembly at an internally generated homopoly(methyl methacrylate) (hPMMA)/homopolystyrene (hPS) interface for the formation of hierarchically porous carbon nanosheets. Reproduced with permission from.^47^ Copyright © 2020 American Chemical Society. (C) Use of an emulsion interface to fabricate anisotropic ultrathin mesoporous organosilica nanosheets. Reproduced with permission from.^53^ Copyright © 2023 American Chemical Society. Scale bars: (A) 200 nm (middle) and 50 nm (right); (B) 200 nm (middle) and 500 nm (right); and (C) 500 nm (middle) and 200 nm (right).

Beyond preformed molecular bilayers, soft interface-induced assembly can also occur at internal interfaces generated through phase separation between immiscible polymers.^59^^,^^87^ Kim et al. developed a polymer-polymer interfacial self-assembly strategy for the construction of hierarchically porous carbon nanosheets (Figure 3B).^47^ In this system, homopoly(methyl methacrylate) (hPMMA), homopolystyrene (hPS), PEO-b-PS, and organic-inorganic precursors were initially dissolved in a common solvent. Evaporation of tetrahydrofuran (THF) induced macrophase separation between the immiscible hPMMA and hPS phases. Owing to the balanced compatibility of PEO-b-PS with the two homopolymers, the block-copolymer-rich precursor phase preferentially migrated to the hPMMA/hPS interface and spread into a nanometer-thick layer to reduce the interfacial energy. The polymer-polymer interface therefore governed the 2D morphology, whereas block-copolymer microphase separation and co-assembly with the precursors generated the ordered mesostructure. Removal of hPMMA and hPS released the freestanding nanosheets, followed by carbonization and selective etching to produce hierarchical micropores and mesopores.

Whereas the polymer-polymer interface described above emerges internally through solvent-evaporation-induced phase separation, a pre-existing interface between two immiscible liquids can likewise provide soft confinement for the formation of 2D mesoporous structures. For example, the research group of Ulrich Wiesner reported the synthesis of ultrathin mSiO_2_ films at the interface between two immiscible solvents.^44^ To enable the growth of 2D superstructures under confined conditions and to obtain a large liquid-liquid interfacial area, a large amount of the oil phase was vigorously stirred and dispersed in an equal volume of the aqueous phase, forming large oil droplets. Furthermore, the research groups of Dongyuan Zhao at Fudan University and Zaiwang Zhao at Inner Mongolia University also reported a double-emulsion-directed assembly strategy to fabricate ultrathin asymmetric mesoporous organosilica nanosheets (Figure 3C).^53^ These nanosheets exhibit anisotropy on their two sides, featuring unique half-open “U-shaped” mesopores. In this system, 1,3,5-trimethylbenzene (TMB) forms large spherical emulsion droplets, while TMB-swollen single micelles combine with organosilica oligomers to generate composite micelles, which are arranged on the surface of the large spherical droplets. Subsequently, the composite micelles rupture on the side adjacent to the large droplets, leading to the formation of the distinctive anisotropic structure.

In summary, by leveraging flexible interfacial confinement, these “soft” interfacial templates offer a versatile and facile platform for the precision synthesis of 2D mesoporous materials, enabling structural control across diverse material systems.

### Directed assembly strategy

Directed assembly is defined here as an assembly route in which the 2D architecture is not established by localizing the pore-forming units at a functionally distinct, spatially extended interface. Instead, the anisotropic morphology emerges from the intrinsic organization or transformation of the assembling species, or from molecular and kinetic effects acting directly on the growing framework. For example, Jiang et al. proposed a molecularly confined single-micelle self-assembly strategy to fabricate 2D mesoporous noble-metal Ir nanosheets (Figure 4A).^39^ PEO-b-PS micelles were used to generate the mesopores, while formic acid acted as both a reducing agent and a morphology-confining agent. The authors proposed that CO generated during the decomposition of formic acid selectively adsorbs on the Ir(111) facets and suppresses out-of-plane crystal growth, thereby favoring the formation of a 2D morphology. This interpretation is supported by control experiments showing that CO generation is required for nanosheet formation. Another directed assembly pathway is based on the formation and preservation of lamellar organic-inorganic composite micelles. Zu et al. utilized lamellar micelles to synthesize a series of 2D Ir-based nanosheets (Ir-IrO_*x*_/C nanosheets) with highly ordered interlayer channels through a nanoconfined self-assembly strategy (Figure 4B).^49^ In contrast to this lamella-preserving pathway, a different directed assembly mechanism involves the *in situ* transformation of lamellar micelles into cylindrical pore-forming domains. Recently, the research group of Dongyuan Zhao and Tiancong Zhao reported the fabrication of 2D mesoporous silica nanodisks with a chiral mesotopography (Figure 4C).^67^ Surfactant molecules initially form lamellar micelles in solution, onto which partially hydrolyzed silanes deposit to form a transient 2D hybrid lamellar intermediate. Subsequently, the cylindrical micelles reassemble within the confined 2D space in the most space-efficient arrangement to maximize occupancy. After removal of the surfactant, these cylindrical micelles generate the final chiral mesopores. Because the initial lamellar micelles both establish the transient 2D precursor and transform into the pore-forming cylindrical micelles, rather than acting as an independent interface separate from the porogen, this route is classified as directed assembly.

**Figure 4:**
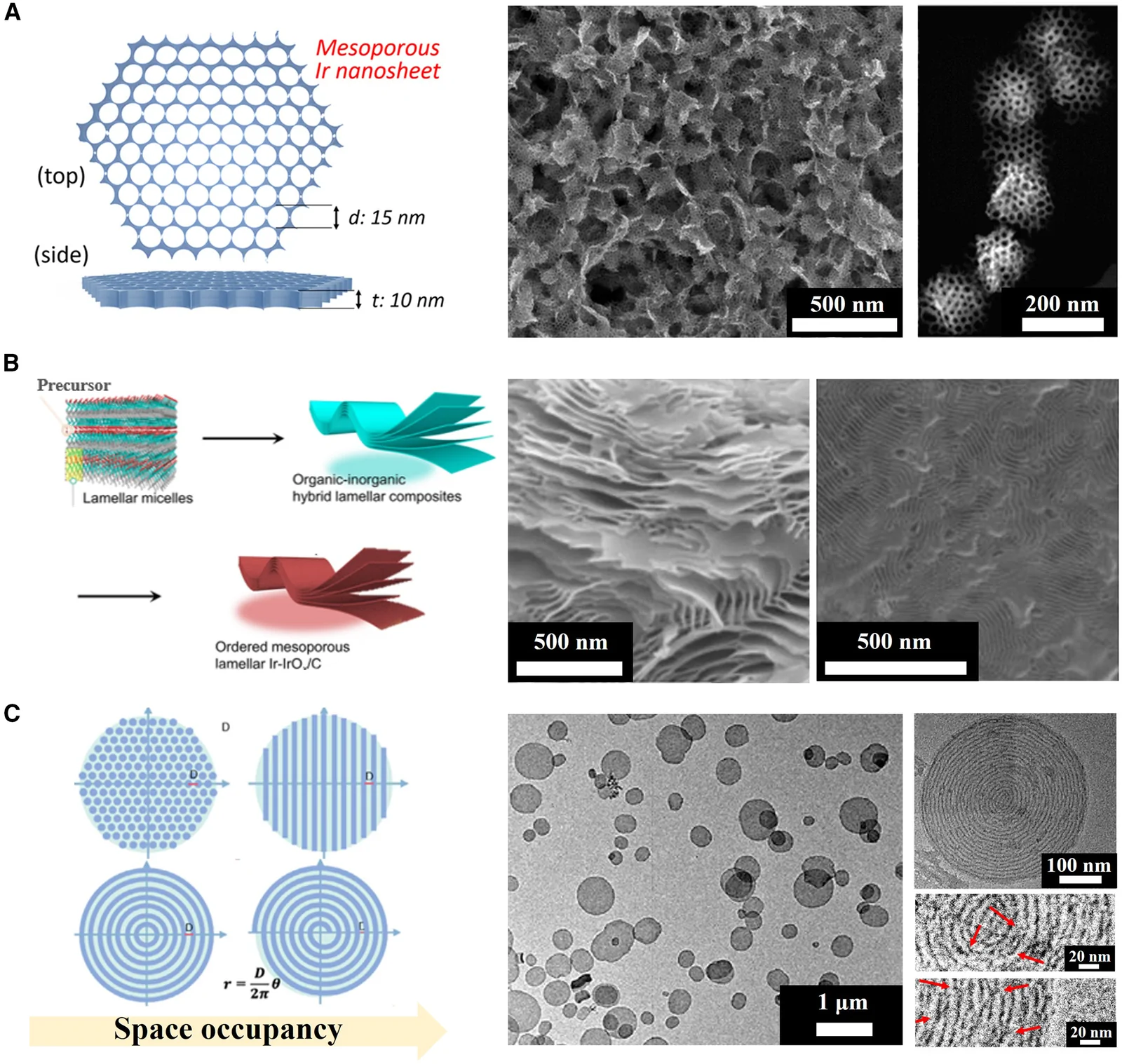
Synthesis of various 2D mesoporous materials via directed assembly strategies (A) Gas-molecule confinement for the formation of mesoporous Ir nanosheets. Reproduced with permission from.^39^ Copyright © 2018 American Chemical Society. (B) Using nanoconfined self-assembly of layered micelles to form ordered mesoporous Ir-IrO_*x*_/C nanosheets. Reproduced with permission from.^49^ Copyright © 2022 American Chemical Society. (C) *In situ* rearrangement of lamellar micelles for the formation of chiral mSiO_2_ nanosheets. Reproduced with permission from.^67^ Copyright © 2026 Wiley-VCH GmbH. Scale bars: (A) 500 nm (middle) and 200 nm (right); (B) 500 nm in both images; and (C) 1 μm (middle), 100 nm (upper right), and 20 nm (two lower-right images).

In summary, the development of directed assembly has significantly enriched the synthetic toolbox for 2D mesoporous materials. By synergistically exploiting interactions across molecular to mesoscopic scales, this approach provides a flexible route for constructing 2D architectures without requiring pre-existing substrates. Such strategies not only enable precise engineering of pore environments but also expand the scope of topological control, allowing the creation of diverse geometries ranging from conventional planar nanosheets to more sophisticated higher-order structures.

### Reaction-derived synthesis strategy

Unlike assembly-mediated routes, reaction-derived strategies generate mesopores through selective chemical conversion, removal, gas evolution, decomposition, or structural reconstruction rather than through the ordered organization of pore-forming units.^66^^,^^88^^,^^89^ Importantly, the formation of the 2D morphology and the generation of mesoporosity do not necessarily occur at the same stage. According to their temporal and mechanistic relationship, reaction-derived strategies can therefore be divided into two subclasses: post-synthetic pore formation within preformed 2D hosts, in which only the pores are introduced after establishment of the sheet-like morphology, and *in situ* porous-sheet formation, in which the 2D morphology and porosity emerge through the same coupled reaction pathway without a pre-existing 2D host.^90^^,^^91^

### Post-synthetic pore formation within preformed 2D hosts

In this pathway, the parent 2D material determines the lateral dimensions and thickness of the final product, while chemically distinct or more reactive domains are subsequently removed to produce mesopores. For example, Bu et al. reported an oxidation and post-etching strategy driven by the redox potential difference to synthesize mesoporous Ti_3_C_2_ with uniform pore sizes (Figure 5A).^52^ The mesopores are generated by hydrofluoric acid (HF) etching of TiO_2_ quantum dots, resulting in Ti_3_C_2_ with a high density of active sites. Moreover, selective etching of active metals serves as another etching strategy to develop 2D mesoporous architectures. The research group of Ben Liu reported a 2D mesoporous PtRhPb nanosheet catalyst with an anisotropic structure and clean metal surfaces (Figure 5B).^57^ The synthesis relies on the selective etching of active Pb and/or Rh metals from Pb-rich Pt_0.75_Rh_0.25_Pb intermetallic nanosheets, thereby generating a 2D mesoporous structure without the need for any surfactants.

**Figure 5:**
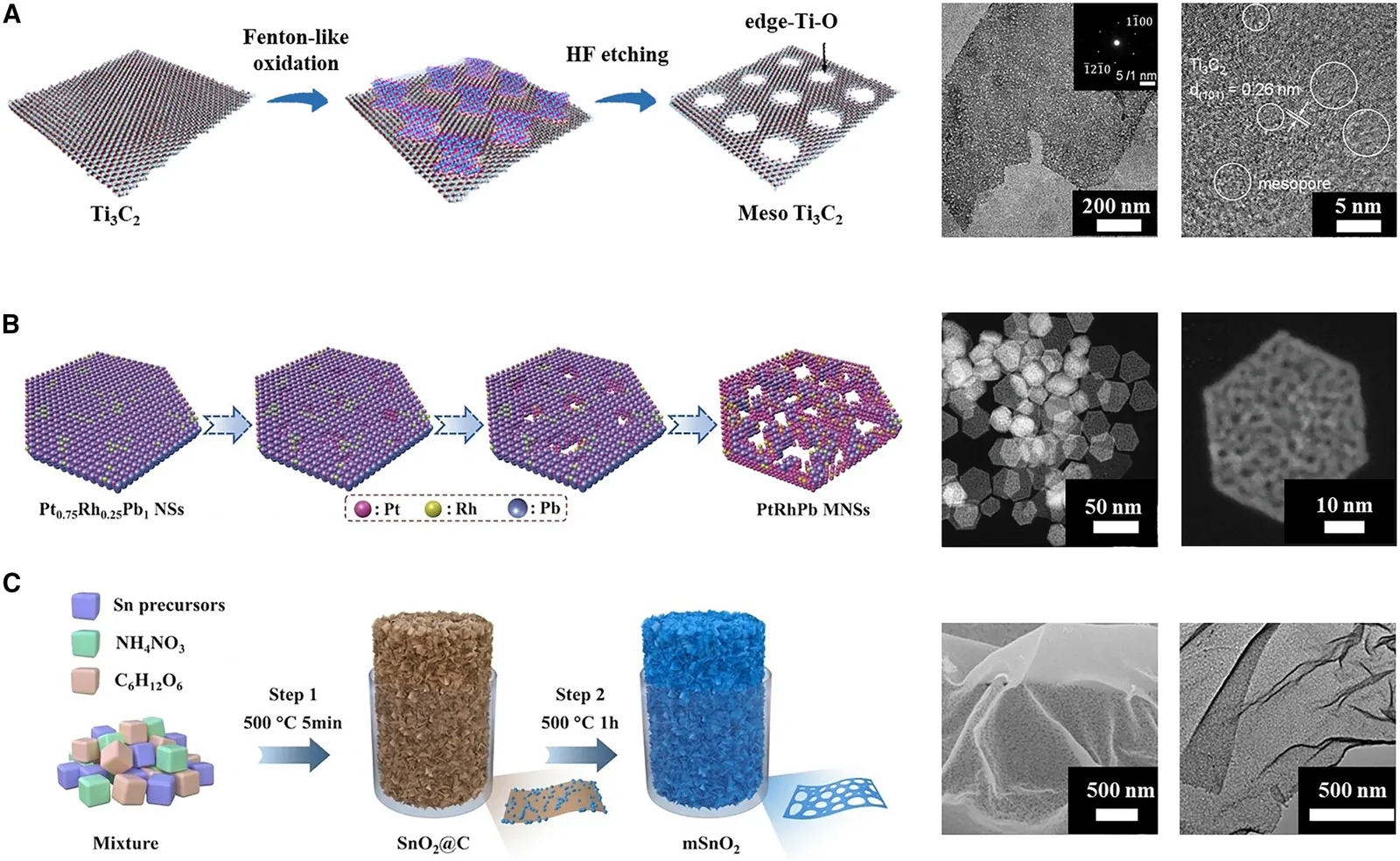
Synthesis of various 2D mesoporous materials via reaction-derived strategies (A and B) Post-synthetic pore formation within preformed 2D hosts. (A) Using an oxidation and subsequent etching strategy driven by redox potential differences to construct mesoporous Ti_3_C_2_ nanosheets. Reproduced with permission from.^45^ Copyright © 2023 American Chemical Society. (B) Using selective etching of active metals to fabricate mesoporous PtRhPb nanosheets. Reproduced with permission from.^82^ Copyright © 2024 Wiley-VCH GmbH. (C) *In situ* porous-sheet formation: using microexplosion reaction to obtain ultrathin 2D mesoporous SnO_2_ nanosheets. Reproduced with permission from.^80^ Copyright © 2024 American Chemical Society. Scale bars: (A) 200 nm (middle) and 5 nm (right); (B) 50 nm (middle) and 10 nm (right); and (C) 500 nm in both images.

Catalytic local decomposition follows the same temporal relationship, although its chemical mechanism differs from dissolution or dealloying. Kim et al. deposited SnO_2_ nanoparticles onto the surface of GO and then heated the composite.^92^ At temperatures below the carbon combustion point, the SnO_2_ nanoparticles catalyzed the selective decomposition of adjacent graphene regions, generating nanopores on the carbon surface that corresponded to the size and distribution of the metal oxide nanoparticles.

### *In situ* porous-sheet formation

In this pathway, no completed 2D host exists before pore generation. Instead, a transient anisotropic intermediate, gas-evolution process, or reaction-induced reconstruction simultaneously directs lateral growth and establishes the porous framework within a coupled synthetic sequence. Jiang et al. reported a rapid synthesis of ultrathin 2D mesoporous SnO_2_ via a microexplosion approach inspired by the Maillard reaction (Figure 5C).^55^ The violent reaction between ammonium nitrate and melanoidin generated rapidly expanding carbonaceous intermediates with a transient 2D morphology. SnO_2_ nanoparticles were anchored on these intermediates and subsequently connected during calcination, while removal of the carbonaceous component exposed the mesoporous oxide framework. Building on this work, this group further fabricated 2D mesoporous In_2_O_3_ nanosheets using the same strategy, where *in situ* generated 2D carbon intermediates serve as sacrificial templates.^64^ Here, “*in situ*” denotes that the morphology and porosity are generated within the same reaction pathway, even though their development may involve sequential elementary steps.

Overall, reaction-derived strategies offer a powerful alternative to traditional assembly mediated routes by bypassing the requirements of ordered co-assembly between surfactants and precursors. Post-synthetic pore formation inherits the geometry of a preformed 2D host and offers relatively independent control over pore generation, whereas *in situ* porous-sheet formation constructs the 2D morphology and porosity simultaneously through coupled reaction kinetics. Such reaction-derived strategies greatly expand the library of 2D mesoporous architectures and compositions, providing a robust and versatile toolkit for the rational design of functional nanostructures beyond the scope of traditional self-assembly.

### Mechanistic principles and structural verification of 2D mesoporous materials

Establishing the synthetic principles of 2D mesoporous materials requires answering two closely related questions: how the anisotropic morphology and mesoporosity are generated, and how the proposed formation pathway and resulting topology can be experimentally verified. The following discussion first examines these governing thermodynamic and kinetic factors and then considers how their structural consequences can be verified using complementary characterization and molecular modeling.

The formation of 2D mesoporous materials can be understood as two coupled but mechanistically distinct processes: the restriction of growth along the out-of-plane direction to establish a 2D morphology and the organization or generation of mesopores within the resulting sheet (Figure 6A). These two processes do not necessarily occur simultaneously. In assembly-mediated routes, the 2D morphology and mesoporosity generally emerge through interfacial selection and micellar organization, followed by removal of the pore-forming agents. In reaction-derived routes, by contrast, the 2D morphology is either inherited from a pre-existing host or generated through a transient anisotropic intermediate, whereas the pores originate from selective etching, gas release, decomposition, or structural reconstruction. Distinguishing the origin of the 2D morphology from that of the mesopores therefore provides a general mechanistic basis for understanding how pore size, orientation, sheet thickness, and structural order can be controlled.

**Figure 6:**
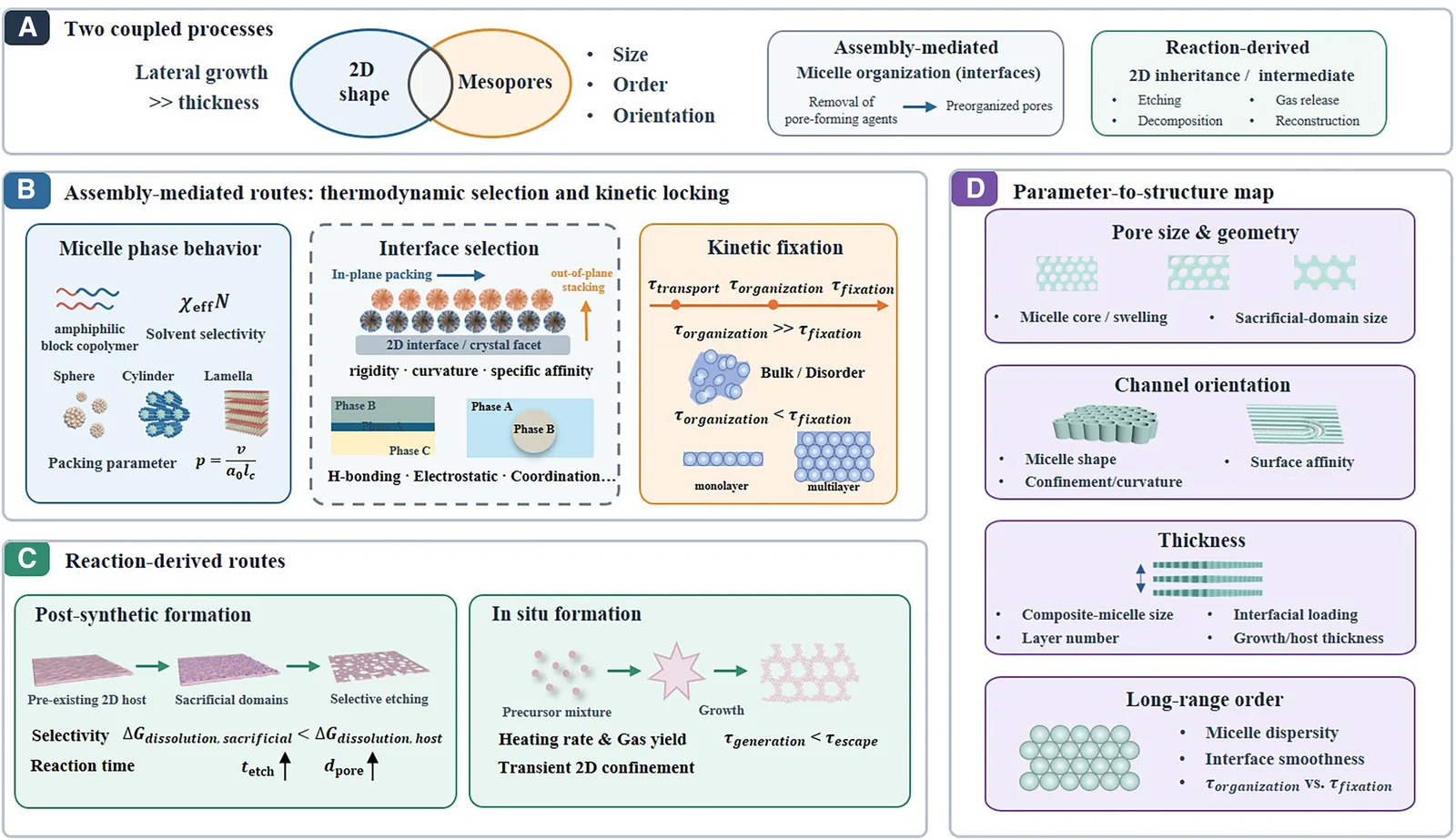
Mechanistic principles governing the formation and structural regulation of 2D mesoporous materials (A) Coupling between 2D morphological restriction and mesopore formation in assembly-mediated and reaction-derived routes. (B) Thermodynamic selection and kinetic fixation governing micellar assembly. (C) Post-synthetic and *in situ* pathways for reaction-derived pore formation. (D) Relationships between synthetic parameters and pore geometry, channel orientation, sheet thickness, and long-range order.

For assembly-generated materials, micellar phase behavior first determines the geometry of the pore-forming domains (Figure 6B). In block-copolymer systems, the effective segregation strength (*χ*_eff_*N*) describes the thermodynamic tendency of chemically dissimilar blocks to undergo microphase separation.^93^ When *χ*_eff_*N* is sufficiently high, unfavorable contacts between the blocks drive the formation of well-defined hydrophilic and hydrophobic domains.^94^ The block volume fraction and molecular asymmetry then determine the preferred interfacial curvature and, consequently, whether spherical, cylindrical, or lamellar domains are favored. In conventional low-molecular-weight surfactant systems, including cationic, anionic, nonionic, and zwitterionic surfactants, for which *χ*_eff_*N* is not an appropriate descriptor, aggregate geometry is governed primarily by molecular packing, surfactant concentration, head group hydration or electrostatic interactions, counterion binding, and solvent conditions. The preferred interfacial curvature arising from the effective molecular geometry is commonly rationalized using the surfactant packing parameter (*p*):

$$p=\frac{v}{a_{0}l_{c}}$$

where *v*, *a*_0_, and *l*_*c*_ are the hydrophobic-chain volume, effective head group area, and chain length, respectively. Increasing *p* generally favors a transition from highly curved spherical micelles to cylindrical and eventually lamellar structures.^6^^,^^95^

Solvent selectivity provides an additional means of regulating micelle size and curvature. A solvent that preferentially solvates one block expands that domain and changes the effective block volume fraction. Selective swelling of the pore-forming core can therefore enlarge the mesopore diameter, whereas increased solvation of the corona may increase the effective head group area and favor more highly curved micelles. Inorganic precursors can further alter this balance through hydrogen bonding, electrostatic attraction, or coordination with the hydrophilic blocks. Thus, the mesopore diameter is not governed solely by the molecular weight of the template, but by the combined effects of block architecture, solvent composition, swelling agents, precursor loading, and precursor-polymer interactions.^94^

Formation of a 2D architecture always additionally requires an interface that energetically favors lateral organization over out-of-plane stacking. A liquid–liquid, gas–liquid, or solid–liquid interface reduces the configurational freedom of the composite micelles and creates an anisotropic environment. The preferred arrangement is determined by the balance among substrate-micelle affinity, micelle-micelle interactions, interfacial rigidity, and curvature. Strong affinity between the micellar domain and the interface lowers the free energy of adsorption and anchors the composite micelles to the interface. The competition between in-plane packing and out-of-plane stacking is especially important for determining mesochannel orientation.^96^ Cylindrical micelles lying parallel to an interface produce channels predominantly parallel to the basal plane, whereas micelles oriented perpendicular to the interface generate pores opening through the basal surface. Parallel alignment is favored when the micelle corona has strong and spatially distributed interactions with the interface, maximizing the contact area. Surface chemistry and precursor condensation can reinforce the selected orientation by immobilizing the micelles before further rearrangement.

This framework also explains why a single composite-micelle layer can form on a salt crystal face instead of unrestricted multilayer stacking.^97^ In representative salt-templated systems, polymer segments or precursor-containing micellar coronas interact strongly with surface ions through coordination, hydrogen bonding, or electrostatic attraction. These specific substrate-polymer interactions preferentially stabilize the first adsorbed micelle layer. After the salt surface is covered, the substrate-specific interaction becomes screened, and additional micelles interact mainly with the previously deposited polymer layer. Because such micelle-micelle interactions are generally weaker than the initial micelle-substrate interaction, the thermodynamic driving force for formation of a second layer is reduced. Moreover, removal of excess solution, limited precursor loading, and rapid solvent evaporation restrict the amount of material available at the interface and kinetically fix the first layer before multilayer growth can proceed. A monolayer therefore results from the combination of preferential first-layer adsorption, finite interfacial loading, and rapid kinetic fixation.

Moreover, the thermodynamic-kinetic balance determines sheet thickness to a certain extent. For a single composite-micelle layer, the minimum thickness is approximately determined by the micelle diameter together with the thickness of the condensed inorganic walls. Larger micelles or more extensively swollen cores therefore increase both the pore diameter and the minimum sheet thickness.^98^^,^^99^ Beyond this intrinsic length scale, the final thickness depends on precursor concentrations, interfacial loading, adsorption time, deposition cycles, condensation rate, and the amount of material supplied during evaporation.^100^^,^^101^ Low loading and rapid fixation favor monolayer or few-layer nanosheets, whereas high loading, prolonged assembly, or slow solidification facilitate multilayer stacking.

Reaction-derived routes follow a different mechanistic pathway (Figure 6C). During post-synthetic formation, a pre-existing 2D host defines the lateral morphology and thickness, while sacrificial domains are selectively removed to generate pores. Pore formation requires a sufficient difference in dissolution or reaction free energy between the sacrificial and framework phases. Increasing the etching time generally increases pore size and pore volume until excessive removal compromises the continuity and mechanical stability of the host.^57^ During *in situ* formation, pores and the 2D morphology can instead arise concurrently through gas evolution, decomposition, or reconstruction. A transient 2D intermediate or anisotropic confinement must persist long enough to suppress three-dimensional growth, while the gas-generation timescale must be shorter than the gas-escape or framework-solidification timescale to preserve the generated voids. Thus, pore size and thickness in reaction-derived systems are controlled primarily by precursor heterogeneity, reaction selectivity, gas-generation rate, mass loss, and reconstruction kinetics rather than by micellar packing.

Overall, the parameter-to-structure map in Figure 6 summarizes how micelle dimensions and sacrificial-domain sizes regulate pore size; micelle geometry, surface affinity, and confinement determine channel orientation; composite-micelle dimensions and interfacial loading affect sheet thickness; and micelle dispersity, interface quality, and organization-fixation kinetics control long-range order.^102^^,^^103^ These relationships are not universal one-parameter laws because changes in solvent composition, precursor chemistry, and interfacial loading frequently affect several structural variables simultaneously. Nevertheless, the combined literature supports a general framework in which 2D mesoporous structures arise from coupled thermodynamic selection and kinetic fixation, thereby connecting synthesis variables to the resulting pore architecture and sheet morphology.

As synthetic strategies for 2D mesoporous materials have become increasingly diverse and structurally sophisticated, the corresponding characterization toolbox has also expanded substantially.^104^ Conventional techniques remain indispensable for establishing the fundamental structural features of these materials. Top-view and cross-sectional transmission electron microscopy (TEM) or scanning electron microscopy (SEM) can directly visualize the lateral morphology, pore arrangement, channel orientation, and overall structural integrity of the nanosheets, while atomic force microscopy (AFM) provides quantitative information on sheet thickness and surface topography. Gas adsorption analysis further evaluates accessible porosity, specific surface area, pore volume, and pore-size distribution, whereas small-angle X-ray scattering (SAXS) and low-angle X-ray diffraction (XRD) reveal characteristic periodicities, pore-to-pore distances, and the degree of mesostructural ordering. When interpreted together, these complementary techniques correlate real-space morphology and pore accessibility with the periodic structural information, thereby providing the fundamental basis for more advanced structural analysis.

Grazing-incidence small-angle X-ray scattering (GISAXS) and grazing-incidence wide-angle X-ray scattering (GIWAXS) provide complementary information on the orientation of 2D mesoporous structures after the nanosheets are deposited, aligned, or assembled on a substrate.^105^ In GIWAXS, the scattering vector is resolved into in-plane and out-of-plane components, allowing the crystallographic texture of the pore-wall framework to be determined.^106^ GIWAXS can establish whether the basal planes of nanosheets lie parallel to the support, whether the crystalline framework adopts a face-on or edge-on orientation, and whether this texture is retained after thermal or chemical treatment. By comparison, GISAXS probes the mesoscopic architecture, including pore orientation, periodicity, symmetry, and anisotropic deformation.^107^^,^^108^ In mesoporous TiO_2_ films, for example, GIWAXS resolved the preferred orientation of the anatase framework, while *in situ* GISAXS followed the organization and fusion of F127-derived micelles into vertically accessible nanopores.^109^ Three-dimensional and *in situ* GISAXS have likewise been used to quantify pore deformation in ordered silica films and to track the development of oriented mesopores during Pt electrodeposition.^110^^,^^111^

Precise X-ray photoelectron spectroscopy (XPS) analysis is particularly important for 2D mesoporous materials because their high surface exposure makes their properties highly sensitive to oxidation states, surface defects, adsorbates, and charging effects.^112^ Reliable interpretation requires appropriate energy calibration and transparent reporting of the background, line shape, full width at half maximum, spin-orbit splitting, branching ratio, and fitting constraints.^113^ Beyond careful peak fitting, XPS should be combined with complementary techniques to achieve more reliable identification of surface states and active sites. Electron paramagnetic resonance (EPR) can probe paramagnetic vacancies, X-ray absorption near-edge structure (XANES) can determine average oxidation states, extended X-ray absorption fine structure (EXAFS) can reveal local coordination numbers and bond distances, and operando spectroscopy can track chemical-state evolution under catalytic or electrochemical conditions. For fully exposed Pt clusters on ordered mesoporous TiO_2_ nanosheets, XPS was complemented by aberration-corrected high-angle annular dark-field scanning transmission electron microscopy (AC-HAADF-STEM) and Pt L_3_-edge XANES/EXAFS, allowing the oxidation state, atomic dispersion, and low average Pt-Pt coordination number to be evaluated together.^114^ Similarly, mixed Ir/IrO_*x*_ sites in ordered nanosheet assemblies were identified through the combined use of XPS, XANES, EXAFS, EPR, and electron microscopy.^49^

Molecular modeling is an important tool for examining assembly pathways and testing proposed structural configurations in 2D mesoporous materials.^115^ Atomistic molecular dynamics (MD) can quantify precursor–template interactions, solvent distributions, micelle deformation, interfacial adsorption, and the energetic preference for in-plane over out-of-plane assembly. Coarse-grained MD (CGMD) and dissipative particle dynamics (DPD) extend the accessible length and time scales, enabling comparisons among spherical, cylindrical, lamellar, and surface-confined micellar states.^116^^,^^117^ Simulations of nonionic surfactants on graphene have shown how surface affinity and surfactant concentration regulate interfacial aggregate morphology, while CGMD simulations of copolymer-nanoplate systems have revealed selective polymer adsorption and chain organization at 2D interfaces.^118^^,^^119^ Brownian dynamics simulations have reproduced the fusion of seed micelles into 2D disk-like assemblies, providing a kinetic explanation for preferential lateral growth.^120^ MD calculations of solvent-block-copolymer interactions have supported the template-evolution mechanism proposed for ordered mesoporous carbonaceous nanosheets, linking changes in solvent affinity to the transformation of polymer assemblies and the resulting pore topology.^121^ Density functional theory-based geometry optimization can additionally evaluate the relative stability of candidate interfacial configurations, defect structures, and active-site geometries. These calculations are most convincing when their predictions are compared with experimentally measured dimensions, interaction energies, scattering features, or spectroscopic signatures. Nevertheless, obtaining a stable or low-energy geometry from simulation does not by itself prove that this structure exists experimentally. A credible model should therefore generate experimentally testable observables.

Taken together, precise identification of a 2D mesoporous topology should rely on convergent evidence rather than any single characterization result. Within this framework, a proposed driving force or assembly pathway should first be translated into specific and falsifiable structural or temporal signatures. This mechanism–observable–validation framework not only strengthens structural assignment but also distinguishes thermodynamic control from kinetic trapping and identifies the parameters that can be deliberately adjusted to regulate pore size, orientation, thickness, and structural order in future synthetic design.

## Applications enabled by the structural specificity of 2D mesoporous materials

Owing to their highly tunable pore structures, large specific surface areas, and unique dimensional effects, 2D mesoporous materials exhibit distinct advantages across various fields.^122^^,^^123^^,^^124^ Compared with conventional bulk materials, 2D mesoporous materials offer faster molecular diffusion channels and greater exposure of active sites, significantly enhancing performance in various areas such as catalysis, energy storage, sensing, and biomedicine.^125^^,^^126^^,^^127^ Beyond these fundamental attributes, the design of higher-order topologies and integration into continuous frameworks further expands their functional capabilities.^128^^,^^129^^,^^130^^,^^131^ Rather than grouping applications solely according to conventional end-use fields, the following discussion is organized according to the characteristic length scales and dominant processes through which 2D mesoporous architectures generate functional advantages (Figure 7). This framework encompasses three complementary levels: (1) individual nanosheets that accelerate physicochemical interfacial kinetics by regulating molecular, ionic, and electronic transport; (2) nano–bio interfaces at which morphology, mesoporosity, topology, and curvature influence biological barrier navigation; and (3) integrated materials and devices in which nanosheets form continuous porous networks for mass and charge transport. These levels are not intended as sequential stages, and a given material may contribute at more than one level depending on its mode of organization and application. By linking structural features and formation mechanisms to material functions, this perspective provides a common basis for discussing catalysis, electrochemistry, energy storage, biomedicine, membranes, electrodes, and sensing.

**Figure 7:**
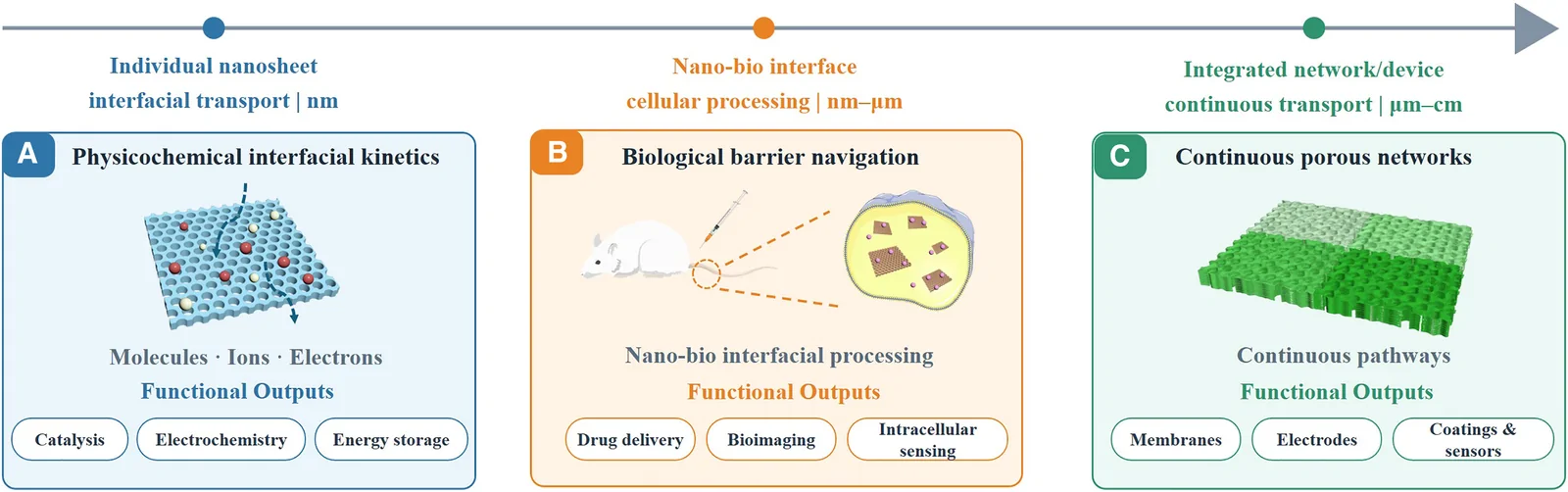
Characteristic length scales and functional outputs of 2D mesoporous materials

### Enhancing interfacial kinetics through 2D mesoporous architectures

At the most fundamental level, the ultrathin and mesoporous nature of 2D architectures serves as a robust structural basis for accelerated interfacial processes.^132^^,^^133^ By combining short diffusion pathways with expansive surface areas and fully accessible active sites, these materials allow reactants to rapidly reach catalytically or electrochemically relevant regions. Moreover, the coupled two-dimensional and mesoporous framework enables efficient transport of electrons, ions, and molecules, thereby mitigating kinetic limitations associated with mass and charge transfer.^134^ These features make 2D mesoporous materials particularly attractive for applications where the rate of interfacial processes critically governs overall performance.^135^^,^^136^

Catalysis most directly benefits from the accelerated interfacial kinetics enabled by 2D mesoporous architectures, as catalytic performance is intrinsically governed by the accessibility of active sites and the efficiency of mass and charge transport across the interface.^51^^,^^60^^,^^137^ In conventional organic catalysis, where rapid substrate diffusion and repeated access to catalytic sites are essential, Zhang et al. synthesized 2D mesoporous polydiaminopyridine (MPDAP) as a high-performance catalyst for Knoevenagel condensation reactions.^138^ Using benzaldehyde and malononitrile as the model substrates, the reaction catalyzed by MPDAP-2 in toluene under air at 80 °C followed apparent first-order kinetics, with a rate constant of 0.380 h^−1^. After 12 h, MPDAP-2 achieved 96% conversion with 99% selectivity and maintained high conversion and selectivity over five consecutive cycles. These results were attributed to the accessible basic nitrogen sites and shortened molecular transport pathways provided by its open mesoporous channels and ultrathin geometry. Beyond molecular accessibility, 2D mesoporous architectures are also highly advantageous for photocatalysis, where efficient light utilization must be coupled with rapid separation and migration of photogenerated charge carriers. Chang et al. successfully constructed 2D mesoporous TiO_2_ nanosheets (mTSs) via a single-micelle-directed interfacial assembly strategy, followed by the loading of uniformly dispersed ultrasmall Pd_0.5_Pt_1_ bimetallic nanoclusters (Figure 8A).^139^ In this system, the 2D mesoporous framework shortens transport pathways and maximizes exposed reaction interfaces, while oxygen vacancies and bimetallic cocatalysts synergistically facilitate charge separation and suppress carrier recombination. Under irradiation from a 300 W Xe lamp with a light intensity of 0.6 W cm^−2^, mTSs-Pd_0.5_Pt_1_ achieved a catalyst-mass-normalized H_2_ evolution rate of 17.4 mmol h^−1^ g^−1^ using 25 mg of catalyst dispersed in 50 mL of water containing 20 vol % triethanolamine as the sacrificial agent (Figure 8B). Under the same experimental conditions, it outperformed the unmodified mTSs and the corresponding monometallic and compositionally varied Pd-Pt controls, supporting the synergistic contributions of the mesoporous nanosheet architecture, oxygen vacancies, and bimetallic cocatalysts. The same structural advantages become even more pronounced in electrocatalysis, where catalytic performance is determined by the coupled kinetics of charge transfer, intermediate adsorption, and surface redox conversion. Wang et al. reported a template-free synthetic strategy for preparing uniform, high-purity 2D single-crystalline mesoporous high-entropy oxide (SC-MHEO) nanoplates (Figure 8C).^140^ When applied to the 5-hydroxymethylfurfural oxidation reaction (HMFOR) for biomass upgrading, SC-MHEO achieved 99.3% HMF conversion and a 97.7% Faradaic efficiency for 2,5-furandicarboxylic acid (FDCA) in 1.0 M KOH containing 10 mM HMF (Figure 8D). Under the same conditions, these values were substantially higher than the 87.2% conversion and 84.9% FDCA Faradaic efficiency obtained with particle-like high-entropy oxide (P-HEO) featuring an irregular nanostructure. These results demonstrate that the single-crystalline mesoporous framework with a high-entropy composition can effectively accelerate interfacial charge transfer and optimize surface reaction pathways, thereby enabling highly selective electrocatalytic conversion.

**Figure 8:**
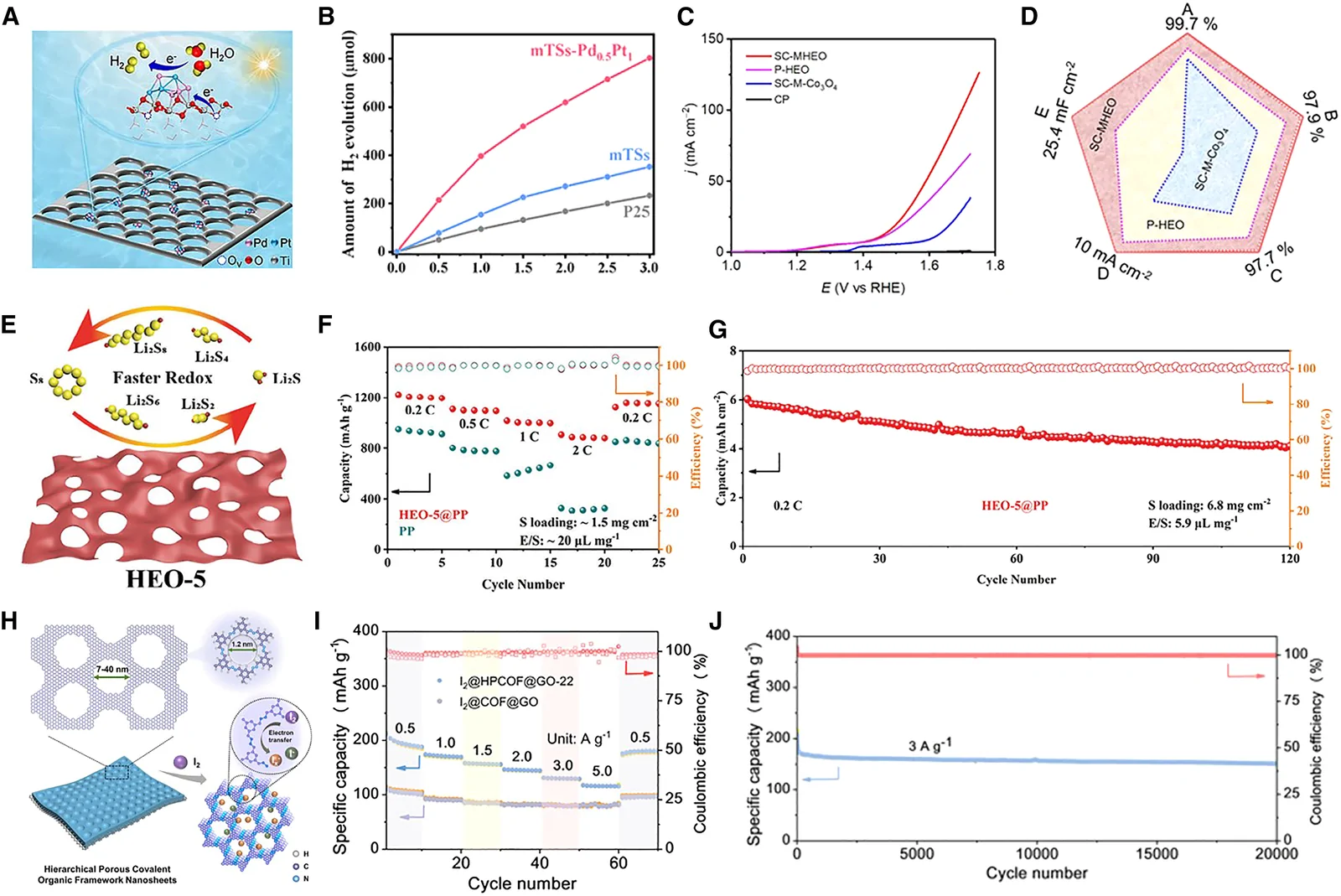
2D mesoporous architectures for enhanced active-site accessibility and interfacial reaction kinetics (A and B) Oxygen vacancy-rich 2D mesoporous TiO_2_ nanosheets integrated with bimetallic nanoclusters for photocatalytic hydrogen evolution: (A) schematic illustration and (B) time courses of photocatalytic H_2_ evolution. Reproduced with permission from.^139^ Copyright © 2025 Wiley-VCH GmbH. (C and D) 2D mesoporous high-entropy oxide (SC-MHEO) nanoplates for electrocatalytic 5-hydroxymethylfurfural oxidation reaction (HMFOR): (C) linear sweep voltammetry (LSV) curves and (D) comparison of key HMFOR performance parameters. Reproduced with permission from.^140^ Copyright © 2024 The Author(s). (E–G) Mesoporous metal oxide nanosheets as catalysts for lithium-sulfur (Li-S) batteries: (E) schematic illustration of the catalytic effect of LaFe_0.4_Co_0.2_Ni_0.2_Cu_0.2_O_3_ (HEO-5), (F) rate capability of different cells, and (G) cycling performance of HEO-5@pristine polypropylene cell with 6.8 mg cm^−2^ S loading. Reproduced with permission from.^54^ Copyright © 2024 Wiley-VCH GmbH. (H–J) Hierarchically porous COF-based (HPCOF) nanosheets as cathodes for zinc-iodine batteries: (H) schematic illustration of the synthesis of HPCOF@GO nanosheets, (I) rate capability of I_2_@HPCOF@GO-22 and I_2_@COF@GO electrodes, and (J) cycling performance of I_2_@HPCOF@GO-22 electrodes at 3 A g^−1^. Reproduced with permission from.^56^ Copyright © 2024 Elsevier Inc.

Extending to sustained electrochemical operation, 2D mesoporous materials are also highly promising for energy storage systems, where continuous ion/electron transport and stable interfacial redox processes are essential for both rate capability and long-term durability.^62^^,^^141^ In this context, Li et al. reported honeycomb-like mesoporous Co, N-doped carbon nanosheets (MC-NS) with high surface area and abundant defects, which enable effective polysulfide confinement and rapid conversion.^142^ Both density functional theory calculations and experiments reveal that Co-N-C active sites and carbon defects synergistically suppress the shuttle effect while promoting polysulfide redox. As a result, at an average sulfur loading of 1.5 mg cm^−2^, the MC-NS/S electrode delivered an initial specific capacity of 714 mAh g^−1^ and retained 512 mAh g^−1^ after 1000 cycles at 2 C, corresponding to an average capacity decay of 0.028% per cycle. Under the same conditions, the carbon nanosheet/sulfur control without surface mesopores (C-NS/S) and the carbon nanoparticle/sulfur control (C-NP/S) retained only 270.5 and 192.2 mAh g^−1^, respectively.

Similarly, Wang et al. synthesized a series of mesoporous metal oxide nanosheets (MMONs) with high crystallinity and remarkable porosity (Figure 8E).^54^ Benefiting from their abundant exposed active sites, these materials served as efficient catalytic components in lithium-sulfur (Li-S) batteries. The Li-S cell with mesoporous LaFe_0.4_Co_0.2_Ni_0.2_Cu_0.2_O_3_ nanosheets as catalyst achieved superior ultralong cycling life over 1500 cycles at 2 C with only 0.041% capacity decay per cycle and a high areal capacity reaching 6.0 mAh cm^−2^ at a low electrolyte/sulfur (E/S) ratio of 5.9 μL mg^−1^ (Figures 8F and 8G). Such structure-enabled electrochemical regulation can be generalized beyond sulfur conversion chemistry to other multistep redox storage systems.

Moreover, the advantages of 2D mesoporous architectures extend beyond those observed relative to their bulk counterparts. Introducing mesopores into 2D metal-organic frameworks (MOFs) and covalent organic frameworks (COFs) can improve guest transport and increase the accessibility of internal active sites. In the CO_2_ cycloaddition of the bulky substrate glycidyl-2-methylphenyl ether, conducted using 20 mg of catalyst and 25 mg of tetrabutylammonium bromide (TBAB) in *N*,*N*-dimethylformamide (DMF) at 65 °C under 5 bar of CO_2_ for 10 h, 2D UiO-66-NH_2_ hierarchically porous nanosheets afforded a cyclic carbonate yield of 96%. Under identical conditions, mesoporous UiO-66-NH_2_ crystals and conventional microporous UiO-66-NH_2_ crystals produced yields of 69% and 28%, respectively.^58^ This stepwise improvement is consistent with the combined benefits of accessible mesopores and the shortened transport pathways provided by the 2D nanosheet architecture, which alleviate pore-entry and diffusion limitations for bulky substrates. Halder et al. further showed that converting a 2D triazine-containing polyimide-based covalent organic framework (TP-COF) into porous N-doped carbon nanosheets (TP-COF-C700) improved its electrocatalytic performance in the hydrogen evolution reaction.^143^ In a three-electrode configuration using 1.0 M KOH, TP-COF-C700 required an overpotential of approximately 95 mV to reach 10 mA cm^−2^, compared with 182 mV for the parent TP-COF and 55 mV for commercial 20 wt % Pt/C. Its charge-transfer resistance was also reduced from 94.5 Ω for TP-COF to 28.3 Ω. The improvement was attributed to the continuous conductive carbon network formed during carbonization, together with better pore accessibility and faster interfacial charge transfer. Huang et al. developed a polymerization-induced co-assembly strategy to fabricate 2D hierarchically porous COF-based nanosheets (HPCOF@GO) with intrinsic microporosity and tunable mesopore sizes (Figure 8H).^56^ This hierarchical pore architecture enables strong adsorption of iodide species while simultaneously facilitating their diffusion and interfacial redox conversion. Consequently, in Zn-I_2_ cells using a Zn-foil anode and 2 M ZnSO_4_ electrolyte, I_2_@HPCOF@GO exhibited a satisfactory rate performance at different current densities (191.2 mAh·g^−1^ at 0.5 A g^−1^), significantly higher than that of I_2_@COF@GO, and also demonstrated excellent cycling stability at a high current density of 3.0 A g^−1^, delivering 154.8 mAh g^−1^ after 20,000 cycles (Figures 8I and 8J).

Collectively, these examples demonstrate that the principal advantage of 2D mesoporous architectures lies not merely in increasing surface area, but in fundamentally reconfiguring the kinetics of interfacial processes across diverse reaction environments. By simultaneously optimizing molecular accessibility, charge transport, and active-site exposure, these materials provide a versatile structural platform for high-performance interface-dominated applications.

### Navigating biological barriers through topology engineering

The introduction of topological complexity and curvature into 2D mesoporous materials creates additional levels of functional control.^144^ These geometric features influence interactions with biological interfaces, including protein adsorption and cellular uptake, enabling applications in drug delivery, bioimaging, and intracellular sensing.^145^^,^^146^

At the fundamental level, the superior biological performance of 2D mesoporous scaffolds is dictated by the synergy between their unique planar morphology and open porous frameworks.^48^^,^^147^ Existing studies have shown that the high aspect ratio of 2D nanostructures fundamentally alters the nano-bio interface; specifically, membrane tension and receptor density trigger a distinctive self-rotation mechanism during cellular internalization.^148^ This process, characterized by the formation of flat vesicle-enclosed structures, significantly reduces the internalization distance and accelerates cellular uptake compared to other nanomaterials. As a subclass of 2D nanomaterials, 2D mesoporous materials effectively capitalize on these dimensional advantages while offering an exceptionally high accessible surface area for cargo immobilization. For instance, Chen et al. fabricated biodegradable 2D mesoporous organosilica nanosheets (MONSs) capable of co-loading doxorubicin and ultrasmall CuS nanoparticles.^149^ Benefiting from its ultrahigh specific surface area and porous architecture, the nanosheet achieved a doxorubicin loading capacity as high as 859 μg mg^−1^. More importantly, compared with conventional spherical mesoporous organosilica nanoparticles, the MONSs exhibited not only faster degradation but also more rapid internalization and higher cellular uptake efficiency, owing to their large aspect ratio and distinct cellular entry pathways associated with 2D nanostructures. Yin et al. further demonstrated how a 2D mesoporous architecture can integrate cargo storage and stimulus-responsive therapy within a single nanoplatform.^48^ They coated ultrathin silicene nanosheets with a mesoporous silica shell to construct core/shell-structured Silicene@Silica nanosheets (Figure 9A). The accessible mesoporous shell served as a reservoir for the hypoxia-activated prodrug AQ4N, achieving a loading capacity of up to 43.23 wt % at an AQ4N/carrier feeding ratio of 3.2 (Figure 9B). Meanwhile, the silicene core retained its NIR-II photothermal activity. Upon 1064 nm irradiation, the resulting local hyperthermia not only directly ablated tumor tissue but also aggravated tumor hypoxia, thereby activating AQ4N and coupling photothermal therapy with hypoxia-activated chemotherapy. *In vivo*, the Silicene@Silica-AQ4N plus laser group showed near-complete suppression of tumor growth over the observation period, outperforming either therapeutic component alone (Figure 9C). This system illustrates how 2D geometry and mesoporosity can jointly provide high interfacial accessibility, cargo-loading capacity, and functional integration within an ultrathin therapeutic platform.

**Figure 9:**
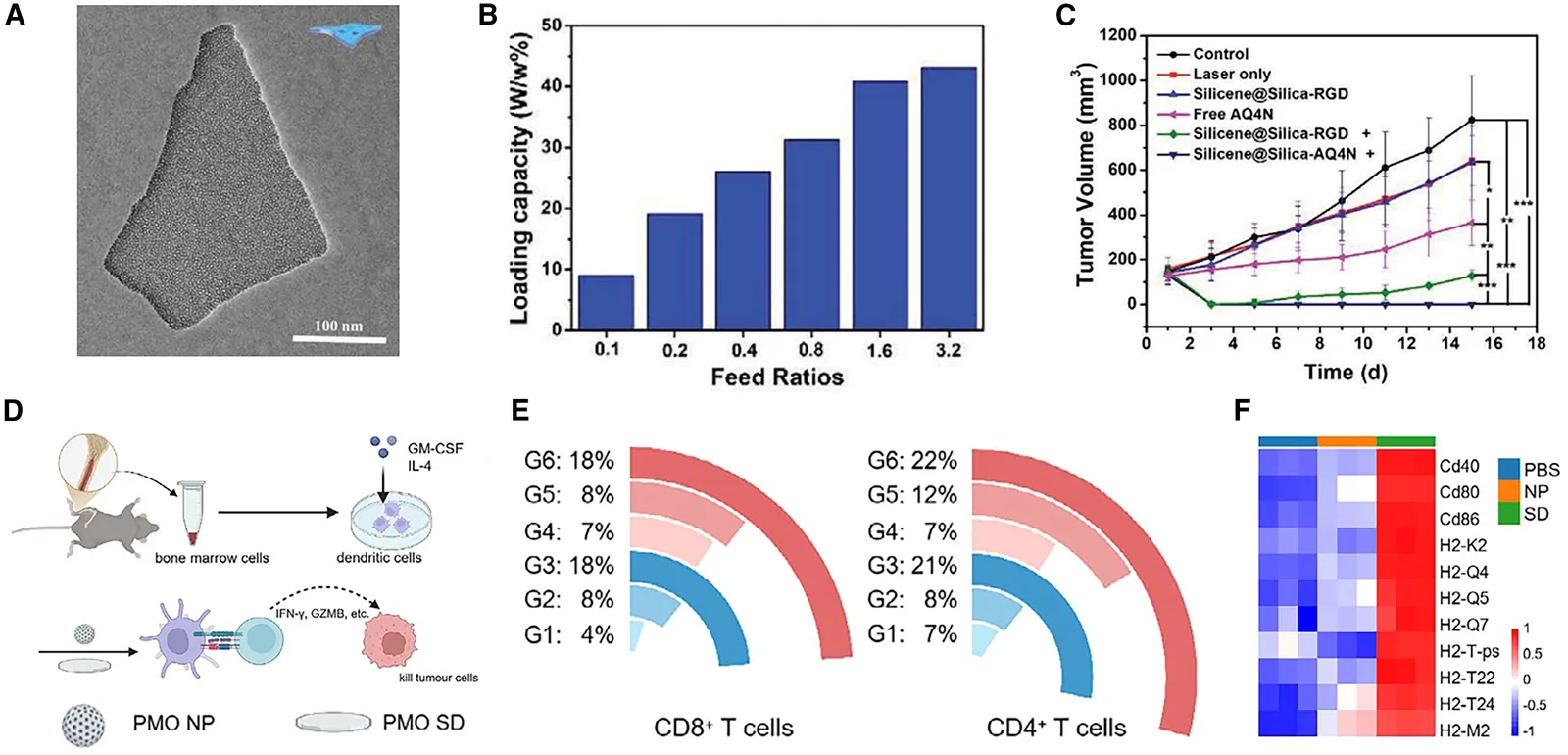
2D mesoporous architectures for navigating biological barriers through topology engineering (A–C) 2D core/shell-structured mesoporous Silicene@Silica nanosheets for synergistic tumor therapy: (A) TEM image showing the 2D morphology and abundant mesopores of the Silicene@Silica nanosheets, (B) AQ4N-loading capacities at different feeding ratios, (C) time-dependent tumor growth after different treatments. Reproduced with permission from.^48^ Copyright *©* 2021 Wiley-VCH GmbH. (D–F) 2D chiral periodic mesoporous organosilica (PMO) nanodisks for vaccine adjuvant applications: (D) schematic illustration of mouse bone marrow dendritic cells (BMDCs) induction, maturation, T cell activation, and antigen-specific tumor-cell killing, (E) activation of CD8^+^ and CD4^+^ T cells by BMDCs in different treatment groups, and (F) heatmap of genes associated with BMDC maturation. Reproduced with permission from.^67^ Copyright © 2026 Wiley-VCH GmbH. Scale bars: (A) 100 nm.

In addition to transport-related barriers, recent studies suggest that 2D mesoporous materials can also regulate more sophisticated biological interfaces, particularly those involved in immune recognition and activation. Dong et al. reported uniform periodic mesoporous organosilica (PMO) nanodisks with a 2D chiral meso-topography as immunological adjuvants (Figure 9D).^67^ Treatment of mouse bone marrow dendritic cells (BMDCs) with nanodisks or nanodisks@ovalbumin (nanodisks@OVA) induced approximately 20% CD69^+^CD25^+^ expression in both CD8^+^ and CD4^+^ T cells, significantly exceeding the levels observed for phosphate-buffered saline (PBS), free ovalbumin (OVA), or conventional mesoporous PMO nanoparticles (Figure 9E). Meanwhile, genes associated with BMDC maturation, including CD80, CD86, and multiple major histocompatibility complex (MHC) class II-related molecules, were markedly upregulated in the nanodisk-treated group (Figure 9F).

Beyond their ability to facilitate tissue penetration, cellular internalization, and intracellular transport, several representative 2D mesoporous platforms have shown preliminarily encouraging biosafety profiles under the specific formulations, doses, administration routes, and observation periods examined to date.^48^^,^^150^ Biodegradable mesoporous organosilica nanosheets exhibited low carrier-associated cytotoxicity and favorable short-term tolerance in tumor-bearing mice, together with more rapid framework degradation than their spherical organosilica counterparts. Similarly, mesoporous silica-coated Nb_2_C MXenes showed satisfactory biocompatibility and *in vivo* tolerance after poly(ethylene glycol) (PEG) functionalization and cyclo (Arg-Gly-Asp-D-Tyr-Cys) (c(RGDyC)) conjugation, while retaining efficient tumor accumulation and therapeutic activity.^151^ Encouraging short-term biosafety results have also been reported for mesoporous black phosphorus nanosheets, core/shell-structured mesoporous Silicene@Silica nanosheets, mesoporous silica-coated graphene nanosheets, and WS_2_-based mesoporous nanocomposites, although the scope and duration of the safety evaluations differed substantially among these studies.^48^^,^^152^^,^^153^^,^^154^ Surface engineering through PEGylation, polydopamine modification, peptide conjugation, or the introduction of silica and organosilica shells has been widely used to improve dispersion in physiological media and enhance tumor-targeting or drug-delivery performance.^153^^,^^155^ In addition, several of these platforms also possess composition-dependent degradation pathways: organosilica nanosheets undergo framework disintegration under biologically relevant conditions, mesoporous black phosphorus systems combine degradation of the silica component with oxidative transformation of black phosphorus, and silicene-based nanosheets can gradually transform into soluble silicon-containing products.^156^^,^^157^^,^^158^ Collectively, these studies suggest that appropriately engineered 2D mesoporous materials can provide an acceptable preliminary safety window for drug delivery, bioimaging, and localized cancer therapy.

Overall, the biomedical potential of 2D mesoporous materials is rooted in the distinctive advantages of their planar mesoporous framework, which offers exceptional accessibility for cargo loading and intimate interaction with biological interfaces. Importantly, this foundation can be further elevated by incorporating topological complexity, which introduces new geometric cues for regulating how these materials navigate cellular, vascular, and immunological barriers. From this perspective, topology engineering does not replace the intrinsic merits of 2D mesoporous scaffolds, but rather builds upon them to unlock a broader and more sophisticated biological design space.

### Constructing functional systems through continuous porous networks

At the application level, mesoporous design principles can be extended from discrete sheet-like materials to continuous films, membranes, electrodes, and interfacial coatings that preserve nanoscale thickness, accessible mesoporosity, or directionally organized transport pathways.^159^^,^^160^ Directly formed continuous porous films or membranes, although outside the primary material-level definition adopted in this review, are discussed as adjacent device-level architectures to illustrate how related mesoporous design principles can be translated into integrated functional systems. Techniques such as vacuum filtration and layer-by-layer assembly enable the formation of membranes, electrodes, or interface coatings that maintain open pore networks and directional transport pathways.^161^^,^^162^ Such continuous architectures facilitate ionic, molecular, or electronic transport while retaining the advantages of mesoporous nanosheets, including high surface accessibility and tunable porosity.^163^^,^^164^ These device-level integrations extend the functionality of 2D mesoporous materials beyond single nanosheets, enabling applications in separation technologies, catalytic membranes, flexible electronics, and sensing interfaces.

A representative example is found in nanofluidic sensing, where continuous porous thin films can translate nanoscale channel design into highly sensitive molecular recognition platforms.^165^ Zeng et al. constructed a nanofluidic sensor based on super-assembled mesoporous thin films with asymmetric nanochannels for the ultrasensitive detection and recognition of small molecules.^166^ By introducing an ultrathin tannic acid (TA)-modified mesoporous SiO_2_ layer (TA-mSiO_2_) onto an anodic aluminum oxide (AAO) array, the resulting heterostructure enabled highly sensitive dopamine detection. The device exhibited three linear dopamine-response ranges of 0.001–1, 1–10, and 10–200 μM, with a detection limit of 0.1 nM, and was further validated in fetal bovine serum. Moreover, the sensing performance could be further tuned by optimizing the thickness of the mSiO_2_ layer, underscoring the importance of maintaining continuous and controllable ion transport pathways across the assembled film.

The same structural principle can also be extended to electrochemical energy storage systems, where continuous porous frameworks serve not only as active components but also as ion-regulating transport media. Ai et al. constructed a reliable superstructured solid electrolyte interphase for stable Zn anodes using mesoporous polydopamine (2D-mPDA) platelets as building blocks (Figures 10A and 10B).^167^ The resulting interphase adopted a biomimetic nacre-like “brick-and-mortar” architecture, while preserving hexagonally ordered in-plane mesoporous channels that functioned as artificial transmembrane ion pathways. This continuous superstructured porous interface functioned as an artificial ion-transport network to regulate Zn^2+^ desolvation and flux distribution. In 2D-mPDA@Zn//Cu asymmetric cells using a 2 M ZnSO_4_ electrolyte, the electrode maintained an average Coulombic efficiency of 99.8% over 1500 Zn plating/stripping cycles at a current density of 5 mA cm^−2^ (Figure 10C). Under the same testing conditions, the 2D-mPDA@GO@Zn control, in which the inner GO layer blocks the through-plane mesopores while retaining a similar platelet-like morphology, and the bare Zn electrode exhibited substantially shorter cycling lifetimes. This comparison supports the importance of accessible, ordered mesoporous channels in providing low-tortuosity Zn^2+^ transport pathways. Beyond interphase engineering, continuous porous nanosheet assemblies can also function as separator-level ion-regulating frameworks in aqueous batteries. Wu et al. reported an ultrathin mesoporous ionic brush (MiB) separator constructed from sulfonic acid-grafted ordered mSiO_2_ nanosheets for stable aqueous zinc metal batteries.^68^ Owing to the continuous ion-conducting porous network and the regulated Zn^2+^ transport enabled by the functionalized mesoporous scaffold, the MiB-based Zn||Zn cell delivered stable cycling for over 4300 h at 1 mA cm^−2^ and 1 mAh cm^−2^. The superior performance of the MiB separator was further confirmed in comparison with previously reported systems in terms of separator thickness, current density, and cycling life.

**Figure 10:**
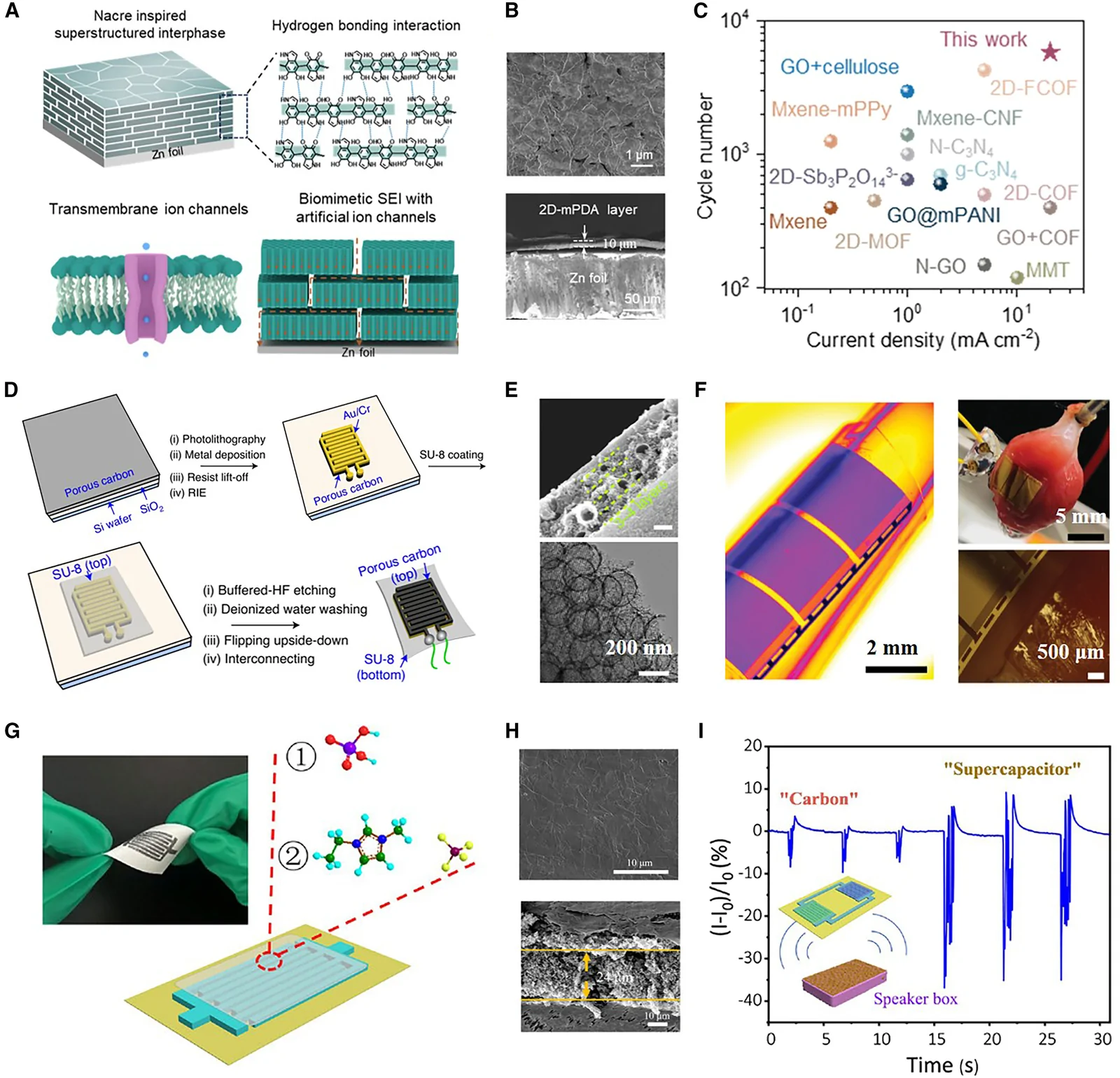
2D mesoporous architectures for constructing functional systems through continuous porous networks (A–C) Reliable superstructured solid electrolyte interphase for stable Zn anodes constructed from mesoporous polydopamine (2D-mPDA) platelets: (A) schematic illustration of the biomimetic superstructured interphase featuring a nacre-inspired “brick-and-mortar” architecture and artificial transmembrane ion channels; (B) top-view and cross-sectional scanning electron microscope (SEM) images of the artificial 2D-mPDA superstructured interphase; (C) performance comparison with previously reported 2D materials used as protective interphases. Reproduced with permission from.^167^ Copyright © 2024 American Chemical Society. (D–F) Porous and monolithic carbon membranes for bioelectronic interfaces: (D) overview of the fabrication workflow for the flexible bioelectronic device; (E) cross-sectional (upper) and top-view (lower) SEM images of the hierarchical porous carbon architecture; (F) photographs showing a micro-supercapacitor-like device conformally interfaced with a cylindrical holder (left) and curvilinear, contractile cardiac tissue (upper right), together with a magnified view of the tissue-device interface (lower right). Reproduced with permission from.^168^ Copyright © 2020, The Author(s), under exclusive licence to Springer Nature Limited. (G–I) In-plane mesoporous N-doped carbon (imNC) for a coplanar integrated microsystem combining a micro-supercapacitor and a pressure sensor: (G) schematic illustration and optical image of imNC-based micro-supercapacitors; (H) cross-sectional and top-view SEM images of the imNC-based microelectrodes; (I) response curves of the integrated micro-supercapacitor-pressure sensor microsystem under acoustic vibrations generated by different spoken words. Reproduced with permission from.^63^ Copyright © 2025 Wiley-VCH GmbH. Scale bars: (B) 1 μm (upper) and 50 μm (lower); (E) 200 nm in both images; (F) 2 mm (left), 5 mm (upper right), and 500 μm (lower right); and (H) 10 μm in both images.

Beyond electrochemical energy storage, the structural merits of continuous 2D porous networks are further extended to the construction of sophisticated bioelectronic interfaces, where simultaneous ionic accessibility, electronic conduction, and mechanical compliance are all required. Fang et al. developed flexible bioelectronic interfaces based on hierarchically porous carbon membranes assembled through a layer-by-layer assembly strategy (Figure 10D).^168^ In this system, ordered mesopores were integrated into a continuous carbon framework and further coupled with larger pores to generate an interconnected porous network throughout the membrane (Figure 10E). Such an architecture allowed electrolytes and biological fluids to readily infiltrate the electrode, thereby greatly enlarging the accessible interface while preserving rapid ion transport across the device. Consequently, the resulting interdigitated microelectrode system exhibited efficient capacitive charge injection and robust electrochemical stability under physiological conditions. Meanwhile, the preserved porous network also improved the mechanical compliance of the membrane, enabling conformal contact with soft biological tissues (Figure 10F).

In more advanced configurations, the continuous porous nature of 2D mesoporous frameworks also enables the integration of multiple device functions into a single flexible platform.^46^^,^^50^ Qin et al. fabricated 2D in-plane mesoporous N-doped carbon (imNC) as a dual-functional material for both micro-supercapacitors (MSCs) and pressure sensors, thereby achieving a co-planar integrated microsystem (Figures 10G and 10H).^63^ Benefiting from the mechanically robust yet porous in-plane framework, the imNC-based MSC-pressure sensor system exhibited sensitive and repeatable responses to bending and torsional vibrations, together with a rapid pressure response (130 ms) and recovery (100 ms). Moreover, when mounted on a speaker, the integrated microsystem was able to reliably detect and distinguish various acoustic signals, such as “carbon” and “supercapacitor” (Figure 10I).

Taken together, these application-oriented examples show that mesoporous design principles can be translated from discrete 2D building blocks to continuous films, interphases, separators, and membranes with collective transport and interfacial functions. Some of these architectures are assembled from preformed 2D mesoporous units, whereas others are formed directly as continuous porous structures. Building on the intrinsic accessibility and tunable porosity of 2D mesoporous units, these continuous architectures enable the translation of nanoscale structural advantages into macroscopic functional performance. In this way, continuous porous network engineering offers a key route for transforming 2D mesoporous materials from discrete nanostructures into integrated functional systems.

### Multiscale structure-property relationships in 2D mesoporous materials

The preceding examples demonstrate that the functional performance of 2D mesoporous materials cannot be attributed to any single structural descriptor, such as surface area, pore size, or sheet thickness. Instead, it emerges from four interconnected structural levels: two-dimensional morphology, mesoporous architecture, pore-wall structure and chemistry, and secondary assembly and integration. To provide an experimental basis for this framework, Table 3 summarizes representative controlled comparisons in which 2D mesoporous materials are evaluated against morphologically, structurally, or compositionally related controls. Although several structural variables remain coupled in some studies, these comparisons reveal recurring links between structural regulation and functional performance.

**Table 3:** Representative controlled comparisons revealing multiscale structure-property relationships in 2D mesoporous materials

Primary structural feature	Representative material	Application	Direct performance comparison	Structure-property relationship	Reference
**Two-dimensional morphology**	hierarchically porous UiO-66-NH_2_ nanosheets	CO_2_ cycloaddition	**cyclic carbonate yield** (hierarchically porous nanosheets vs. mesoporous crystals vs. conventional microporous crystals): 96% vs. 69% vs. 29%	ultrathin 2D geometry and highly open hierarchically porous channels: fast mass transfer and highly accessible Lewis acidic sites	Han et al.^58^
	mesoporous organosilica nanosheets (MONSs)	drug delivery	**cellular uptake** (MONSs vs. spherical organosilica nanoparticles): higher vs. lower; **degradation time** (MONSs vs. spherical organosilica nanoparticles): ∼7 vs. > 14 days;	high aspect ratio and a distinct cellular-internalization pathway: more rapid degradation, higher cellular uptake efficiency, faster internalization	Chen et al.^149^
	chiral mesoporous organosilica nanodisks	immunological adjuvants	**CD69^+^CD25^+^ fractions within CD8^+^ and CD4^+^ T cells** (chiral PMO nanodisks@OVA vs. conventional PMO nanoparticles@OVA): ∼20% vs. ∼10%	2D chiral mesotopography: enhanced immune stimulation	Dong et al.^67^
**Mesoporous architecture**	mesoporous Co,N-doped carbon nanosheets (MC-NS)	Li-S batteries	**capacity after 1000 cycles** (MC-NS vs. non-mesoporous carbon nanosheet vs. carbon nanoparticles): 512 vs. 270.5 vs. 192.2 mAh·g^−1^; **capacity fading rate** (MC-NS vs. non-mesoporous carbon nanosheet vs. carbon nanoparticles): 0.028% vs. 0.062% vs. 0.068% cycle^−1^	high specific surface area and abundant defects: enhanced polysulfide confinement and accelerated redox conversion	Li et al.^142^
	hierarchically porous COF/GO nanosheets (HPCOF@GO)	Zn-I_2_ batteries	**capacity at 0.5 A g**^**−**^**^1^** (I_2_@HPCOF@GO vs. I_2_@COF@GO): 191.2 vs. ∼120 mAh·g^−1^; **capacity after 20,000 cycles** (I_2_@HPCOF@GO vs. I_2_@COF@GO): 154.8 vs. 92.2 mAh·g^−1^	hierarchical micro/mesoporosity: enhanced confinement of iodine species and shortened diffusion pathways	Huang et al.^56^
	2D PtRhPb mesoporous nanosheets (PtRhPb MNSs)	ethanol oxidation electrocatalysis	**mass activity** (PtRhPb MNSs vs. PtRhPb nanoparticles): 7.8 vs. 4.7 A mg^−1^; **C1 product selectivity** (PtRhPb MNSs vs. PtRhPb nanoparticles): 70% vs. 42%	metal mesoporous structure: greater exposure of undercoordinated active sites	Fan et al.^57^
**Pore-wall structure and chemistry**	oxygen-vacancy-rich mTiO_2_–Pd_0.5_Pt_1_ nanosheets	photocatalytic H_2_ production	**H**_**2**_**evolution rate** (mTiO_2_–Pd_0.5_Pt_1_ vs. unmodified mTiO_2_ nanosheets): 17.4 vs. ∼8 mmol h^−1^ g^−1^	oxygen vacancies and Pd-Pt bimetallic cocatalysts: promoted charge separation and suppressed charge-carrier recombination	Chang et al.^139^
	single-crystalline mesoporous high-entropy oxide nanoplates (SC-MHEO)	electrocatalytic biomass upgrading	**HMF conversion** (SC-MHEO vs. particle-like high-entropy oxides vs. single-crystalline mesoporous Co_3_O_4_): 99.3% vs. 87.2% vs. 78.8%; **FDCA Faradaic efficiency** (SC-MHEO vs. particle-like high-entropy oxides vs. single-crystalline mesoporous Co_3_O_4_): 97.7% vs. 84.9% vs. 60.5%	high-entropy composition, mesoporous structure, and single-crystalline phase: increased exposure of electrocatalytically active sites and optimized adsorption/reaction energetics	Wang et al.^140^
**Secondary assembly and macroscopic integration**	Ordered mesoporous polydopamine superstructured interphase (2D-mPDA@Zn)	Zn metal batteries	**nucleation overpotential** (2D-mPDA@Zn vs. pore-blocked 2D-mPDA@GO@Zn vs. bare Zn): 35 vs. 65 vs. 73 mV	hexagonally ordered mesopores: homogenized Zn^2+^ flux, uniform Zn deposition, and suppressed side reactions	Ai et al.^167^
	mesoporous GO nanosheet membrane	molecular separation	**water permeability** (mesoporous-GO membrane vs. pristine-GO membrane): 39.93 ± 0.46 vs. 1.53 ± 0.59 LMH/bar	in-plane mesopores: additional transport channels, shortened average transport distance	Li et al.^169^
	highly ordered monolayer mesoporous TiO_2_ nanomeshes	Sn aqueous-battery anode protection	**cycle life at 1.0 mA cm**^**−**^**^2^** (Sn@meso-TiO_2_ vs. Sn@TiO_2_ nanoparticles): >1400 vs. 90 h; **Charge transfer resistance** (Sn@meso-TiO_2_ vs. Sn@TiO_2_ nanoparticles): 0.5 vs. 2.2 Ω	large specific surface area and highly crystalline ultrathin perforated frameworks: enhanced anti-corrosion ability, accelerated surface interfacial charge transfer, and rapid ion transport	Zhang et al.^170^
	porous MXene nanosheet membrane	H_2_/CO_2_ separation	**H**_**2**_**permeance** (porous-MXene membrane vs. nonporous-MXene membrane): 1335 vs. 419 GPU	tunable in-plane pore: fast mass transfer channels	Wang et al.^171^

The listed structural feature represents the primary descriptor associated with the observed performance trend. In several comparisons, morphology, porosity, crystallinity, and surface chemistry vary concurrently; the proposed structure-property relationships should therefore be interpreted as coupled rather than independently isolated effects. 2D, two-dimensional; C1, one-carbon product; COF, covalent organic framework; HMF, 5-hydroxymethylfurfural; FDCA, 2,5-furandicarboxylic acid; GO, graphene oxide; HPCOF, hierarchically porous covalent organic framework; Li-S, lithium-sulfur; PMO, periodic mesoporous organosilica; Zn-I_2_, zinc-iodine; GPU, gas permeation unit.

Taken together, these comparisons show that structural regulation operates across successive length scales. Two-dimensional morphology governs transport length, site accessibility, and interfacial contact; mesoporous architecture controls guest admission, diffusion, and nanoscale confinement; pore-wall structure and chemistry regulate carrier transport, guest-wall interactions, and active-site reactivity; and secondary assembly and integration determine whether these nanoscale advantages are retained in continuous macroscopic architectures. Figure 11 consolidates these experimentally observed trends into a multiscale structure-property framework and highlights the application-dependent trade-offs among transport, selectivity, reactivity, and structural stability.

**Figure 11:**
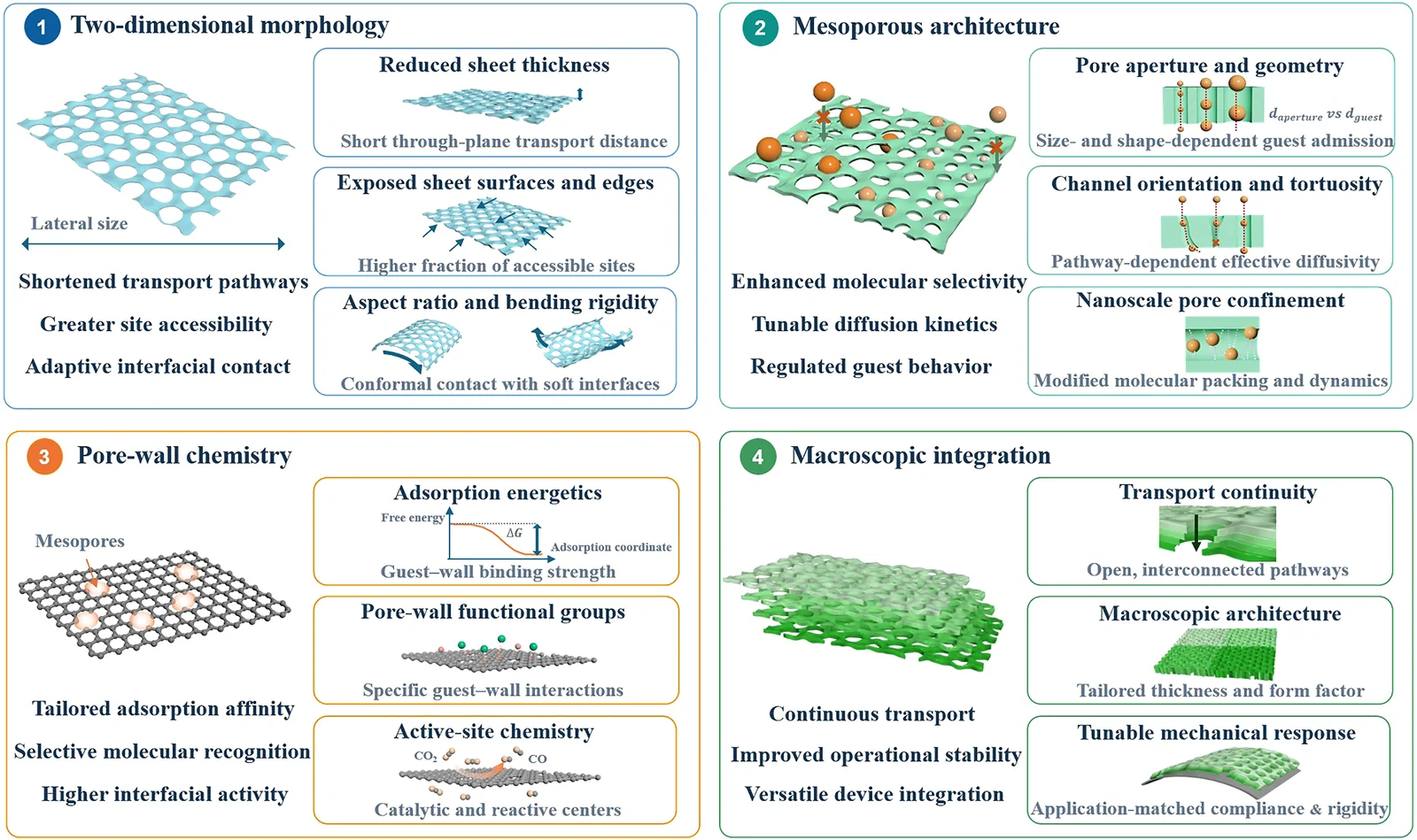
Multiscale structure-property relationships in 2D mesoporous materials

The transport consequences of two-dimensional morphology and mesoporous architecture can be described quantitatively by considering both the characteristic transport length and the effective diffusivity within the porous framework. For diffusion-controlled transport, the characteristic diffusion time can be approximated from Fick’s second law as

$$t_{diff}∼\frac{L^{2}}{D_{eff}}$$

where *L* is the characteristic distance from the external interface to an active site, and *D*_*eff*_ is the effective diffusivity within the mesoporous network.^172^ For nanosheets accessible from both sheet surfaces, the maximum through-thickness diffusion distance is approximately *h*/2, where *h* is the sheet thickness. Therefore, decreasing the thickness from 100 to 10 nm, under otherwise identical conditions, theoretically shortens the characteristic diffusion time by approximately two orders of magnitude. However, this relationship should be regarded as a scaling framework rather than a direct prediction of catalytic rates, because *D*_*eff*_, surface chemistry, active-site density, and the rate-determining elementary step may vary simultaneously.

Mesopore size primarily affects *D*_*eff*_, rather than *L*, by controlling steric restriction, solvation-related confinement, pore filling, and local tortuosity.^173^ This dependence is generally non-monotonic. Mesopores must be sufficiently large to admit solvated ions, reactant molecules, and reaction intermediates, but excessive pore enlargement can reduce surface area, active-site density, and intermediate confinement. Pore size must also be interpreted together with channel orientation, connectivity, and tortuosity. Mesochannels oriented through the basal plane minimize the path from the external surface to the interior, whereas in-plane channels favor lateral transport but can become obstructed when nanosheets restack. Ordered and interconnected channels reduce tortuosity and eliminate isolated dead-end pores, thereby increasing the fraction of surface area that remains dynamically accessible.^174^

Beyond pore geometry, pore-wall structure and chemistry jointly regulate transport, guest-wall interactions, and interfacial reactivity. Where electronic or ionic transport is involved, highly crystalline and continuous walls generally reduce carrier scattering and grain-boundary resistance, whereas polycrystalline or defect-rich walls may impede transport but simultaneously introduce coordinatively unsaturated or reactive sites.^175^ Framework composition, surface functional groups, surface charge, defects, and local coordination environments further determine adsorption energetics, molecular recognition, intermediate stabilization, and active-site reactivity. The optimal guest-wall binding strength is application-dependent: insufficient interactions limit guest uptake and intermediate stabilization, whereas excessively strong binding can hinder product desorption, catalytic turnover, or adsorbent regeneration. Pore-wall design therefore requires balancing carrier transport, adsorption and selectivity, and active-site reactivity rather than independently maximizing crystallinity or binding strength.

At larger length scales, secondary assembly and integration determine whether the intrinsic advantages of individual nanosheets are preserved in practical architectures. Inter-sheet alignment and connectivity can establish continuous molecular, ionic, or electronic pathways, whereas restacking, pore blockage, and poorly connected contacts increase transport resistance and reduce the dynamically accessible surface area. Assembly thickness and form factor further determine device-level transport distances, while inter-sheet coupling controls mechanical integrity, compliance, and conformal contact with rigid or soft interfaces.

These multiscale relationships are strongly coupled and are frequently non-monotonic. Larger pores may facilitate diffusion but reduce surface area or weaken confinement; stronger adsorption may improve capture but hinder release; higher crystallinity may enhance carrier transport while decreasing the density of defect-associated reactive sites; and denser assembly may improve mechanical stability while increasing pore blockage and tortuosity. Reliable structure-property relationships therefore require application-specific optimization and controlled comparisons that isolate individual descriptors while maintaining consistent composition, active-site density, mass loading, testing conditions, and device configuration.

## Conclusions and perspectives

In this review, we have systematically summarized recent progress in the synthesis and application of two-dimensional (2D) mesoporous materials. From a synthetic perspective, the reported approaches can be broadly divided into assembly mediated and reaction-derived strategies. Assembly mediated synthesis includes interface-induced assembly, in which a functionally distinct interface directs the 2D morphology while separate micelles or block copolymers generate the mesopores, and directed assembly, in which the 2D morphology and pore organization arise from the same assembling system or its structural transformation. Reaction-derived synthesis further encompasses post-synthetic pore formation within preformed 2D hosts and *in situ* porous-sheet formation, thereby distinguishing inherited 2D morphology from morphology-porosity co-formation. These complementary routes have enabled increasingly diverse compositions, pore architectures, sheet geometries, and hierarchical structures.

The functional advantages of these materials arise from the coupling between reduced dimensionality and accessible mesoporosity rather than from either feature alone. Ultrathin frameworks shorten transport pathways and expose a large fraction of active sites, while mesopores facilitate the diffusion of molecules and solvated ions that may be excluded from microporous frameworks. Higher-order topology and curvature can further regulate interactions with biological interfaces, whereas the assembly of individual nanosheets into continuous porous networks enables their integration into membranes, electrodes, sensors, and bioelectronic systems. Despite this progress, four closely related challenges continue to limit the transition from case-specific synthesis and proof-of-concept demonstrations toward predictive design and practical implementation (Figure 12).

1. **Synthetic limitations.** The preparation of highly ordered 2D mesoporous materials still relies heavily on specific interfaces, templates, and narrowly optimized assembly conditions. Hard and soft interfaces provide effective suppression of out-of-plane growth, but their compatibility with different precursors is often limited, and their removal or retention can complicate purification and determine whether the final product is a freestanding sheet or a sandwich-like heterostructure. Directed assembly avoids a pre-existing substrate but usually requires the coupled regulation of micellar phase behavior, precursor condensation, solvent composition, and kinetic fixation within a narrow processing window. Reaction-derived routes broaden the accessible compositions but often provide less control over pore uniformity and long-range order. Future research should therefore develop more general intrinsic self-growth and template-free strategies while improving the scalability of established interfacial methods. Reproducibility should be verified by repeating the synthesis under identical conditions and comparing the product yield, pore size, sheet thickness, lateral dimensions, and mesostructural order across different batches. For scale-up evaluation, precursor utilization, processing time, and solvent consumption should also be quantitatively reported. The objective is not simply to eliminate interfaces, but to establish predictable synthetic rules that can be transferred across different compositions and production scales.
2. **Structural control.** Precise and preferably independent control over sheet geometry and mesopore architecture remains difficult because these structural parameters are frequently coupled during synthesis. Changes in template size, solvent selectivity, precursor loading, interfacial affinity, or condensation rate can simultaneously affect pore diameter, wall thickness, sheet thickness, channel orientation, and structural order. Consequently, many reported materials exhibit irregular lateral shapes, broad thickness distributions, disordered mesopores, or partial multilayer stacking. Future studies should move beyond the qualitative formation of sheet-like products toward the deliberate regulation of lateral size, thickness, pore size and distribution, channel orientation, connectivity, wall crystallinity, and defect density. Particular attention should be paid to controlling monolayer versus multilayer growth and to maintaining accessible mesochannels after nanosheet restacking or assembly into macroscopic structures. More sophisticated control over curvature, chirality, asymmetric surfaces, branched geometries, and multilevel pore networks may further expand the functional design space.
3. **Mechanistic understanding.** Many proposed formation mechanisms are still inferred primarily from static images of samples collected at selected stages of synthesis. Such observations cannot reliably distinguish thermodynamically preferred structures from kinetically trapped intermediates or establish the sequence through which micelles, precursors, interfaces, and transient phases evolve. A deeper understanding requires time-resolved identification of the processes that determine in-plane versus out-of-plane packing, monolayer versus multilayer deposition, mesochannel orientation, sheet thickness, and the fixation of transient structures. Following a mechanism-observable-validation framework, each proposed driving force should be translated into experimentally testable structural or temporal signatures, while simulation-derived configurations should be compared with measured dimensions, scattering patterns, interaction energies, or spectroscopic features. Such convergent evidence is essential for transforming empirical synthesis into mechanism-guided structural design.
4. **Quantitative structure-property relationships.** While 2D mesoporous materials have demonstrated clear advantages in a variety of applications, a comprehensive understanding of their structure-property relationships is still lacking, especially concerning their practical implementation in device fabrication. How parameters such as pore size, pore ordering, thickness, defect density, and surface chemistry collectively influence performance requires further systematic investigation.
  In addition, most studies compare 2D mesoporous materials with bulk particles or nonporous counterparts, while direct comparisons with other 2D porous materials, such as 2D MOFs/COFs, under the same testing conditions remain rare. Future studies should therefore use consistent testing methods and carefully control or fully report relevant parameters, including chemical composition, active-site density, pore volume, pore size distribution, nanosheet thickness, mass loading, conductivity, and device configuration. Performance should also be normalized where appropriate. Material stability should be evaluated under application-relevant conditions, including the reaction medium, temperature, and electrochemical potential. Guest size and solvation structure should also be considered in relation to the effective pore aperture and transport kinetics. Such studies would help identify the applications in which each material class performs best and establish more reliable structure-property relationships.
  For biomedical applications, this structure-property framework must explicitly incorporate toxicity and long-term *in vivo* biocompatibility. Although preliminary results are encouraging, biosafety remains a major challenge for the clinical translation of 2D mesoporous materials. Most current evaluations rely on short-term cell viability assays, body-weight monitoring, biochemical measurements, and endpoint histological examination. These tests mainly demonstrate low acute or subacute toxicity under specific experimental conditions, rather than establishing long-term systemic safety. Biological responses may vary substantially with framework composition, anisotropic geometry, lateral size and thickness, aggregation state, cellular uptake, membrane interactions, protein-corona formation, residual surfactants, surface functionalization, and the chemical identity of degradation products. In particular, structural disintegration observed *in vitro* does not necessarily indicate complete degradation and clearance *in vivo*, because intact nanosheets, partially degraded fragments, and soluble products may follow different biodistribution and excretion pathways. Future studies should therefore combine time-resolved biodistribution and mass-balance analyses with multimodal tracking of both intact frameworks and their transformation products. Longer observation periods, repeated-dose experiments, quantitative organ-retention measurements, excretion analysis, and systematic evaluation of chronic inflammation and immune responses will also be required. Importantly, safety criteria should be tailored to the intended application, because intravenously administered nanocarriers, locally injected therapeutic agents, and implanted 2D mesoporous scaffolds differ considerably in exposure route, residence time, degradation requirements, and acceptable risk. Establishing such application-specific and standardized evaluation frameworks will be essential for translating the structural advantages of 2D mesoporous materials into clinically viable technologies.

**Figure 12:**
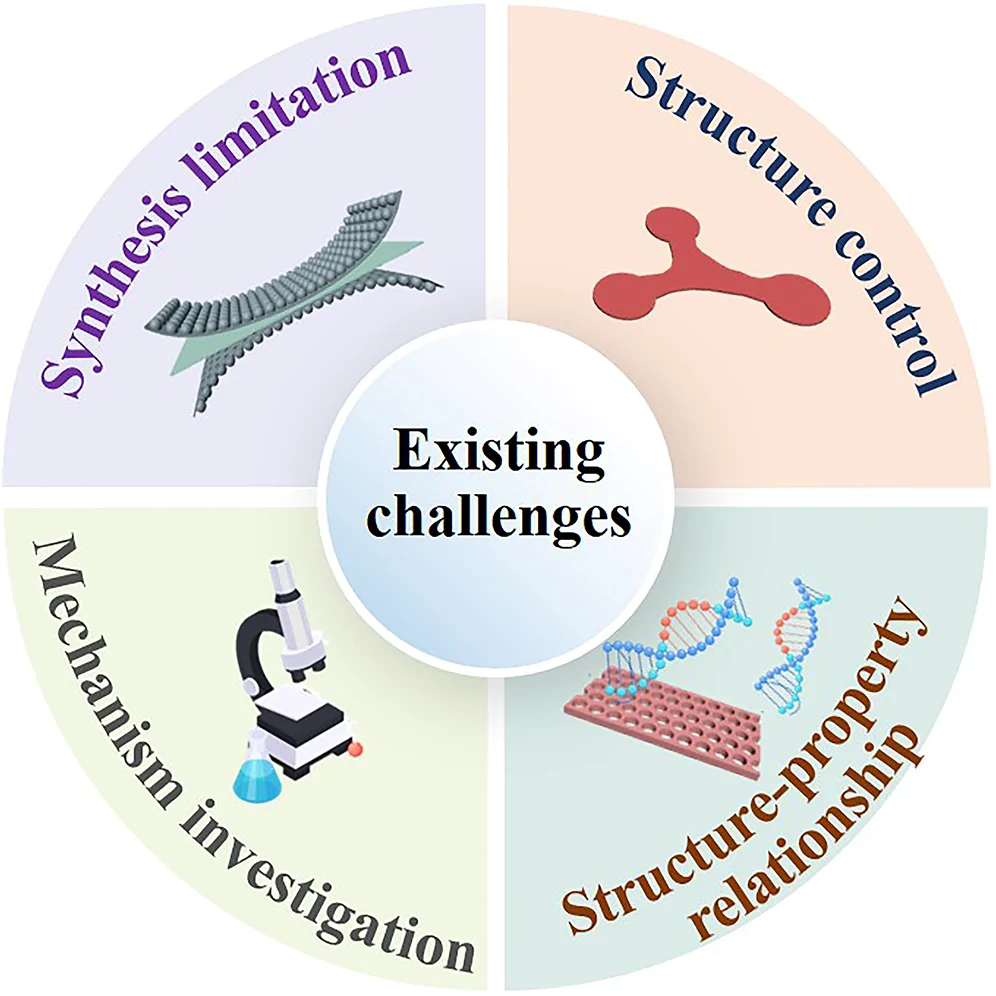
Existing challenges in the development of 2D mesoporous materials

In summary, continued efforts are needed to overcome limitations in synthesis and structural control, deepen mechanistic understanding, and establish robust structure-property relationships. These advances are expected not only to deepen fundamental insights but also to accelerate their practical implementation across multidisciplinary fields.

## Acknowledgments

We gratefully acknowledge funding from the 10.13039/501100001809National Natural Science Foundation of China (22475048, 22305042), Shanghai Rising-Star Program (20QA1401200), and the Fudan-Xiaomi Young Talents Program.

## Author contributions

X.H. conceived the review, conducted the literature survey, prepared the figures and tables, and wrote the original draft. T.Z. supervised the work and reviewed and edited the manuscript.

## Declaration of interests

The authors declare no competing interests.
